# Street level urban metabolism as a tool for mapping urban flows in Amman’s neighborhoods

**DOI:** 10.1038/s41598-025-03821-y

**Published:** 2025-05-28

**Authors:** Anas Tuffaha, Ágnes Sallay

**Affiliations:** Magyar Agrár- és Élettudományi Egyetem, Vaci Ut 10, Budapest, 1322 Hungary

**Keywords:** Street-level urban metabolism, Streetscapes, Green-blue infrastructure, Resource flow analysis, Urban ecology, Environmental impact

## Abstract

This article examines urban systems through the analytical framework of street-level urban metabolism, expanding upon Kennedy’s multi-layered studies of urban metabolic processes. While traditional urban metabolism analyzes cities as macro-scale systems, this study focuses on neighborhood street networks as critical units of resource flow, applying Kennedy’s indicator framework to a finer-grained assessment of streets as metabolic conduits. Focusing on Amman, Jordan, the study dissects seven key metabolic flows (e.g., water, energy, waste) at the street level, adapting methodologies from Rotterdam’s neighborhood-scale metabolic analysis. This approach reveals how hyper-localized flows interact with environmental stressors such as water scarcity, extreme heat, and limited green infrastructure. By comparing four distinct typical neighborhoods in the city, the study identifies disparities in flow efficiency tied to urban design, offering targeted strategies to enhance resilience. The research’s goal is to generate actionable strategies for enhancing urban infrastructure by mapping resource flows and proposing interventions. Ultimately, aiming to develop a scoring system that quantifies the impact of each flow within cities studied, and complemented by a survey to understand the local’s perspective to identify analytically, flows and elements that enhance the city’s metabolism and environmental stresses.

**Supplementary Information:**

The online version contains supplementary material available at 10.1038/s41598-025-03821-y.

## Introduction

**Fig. 1:**
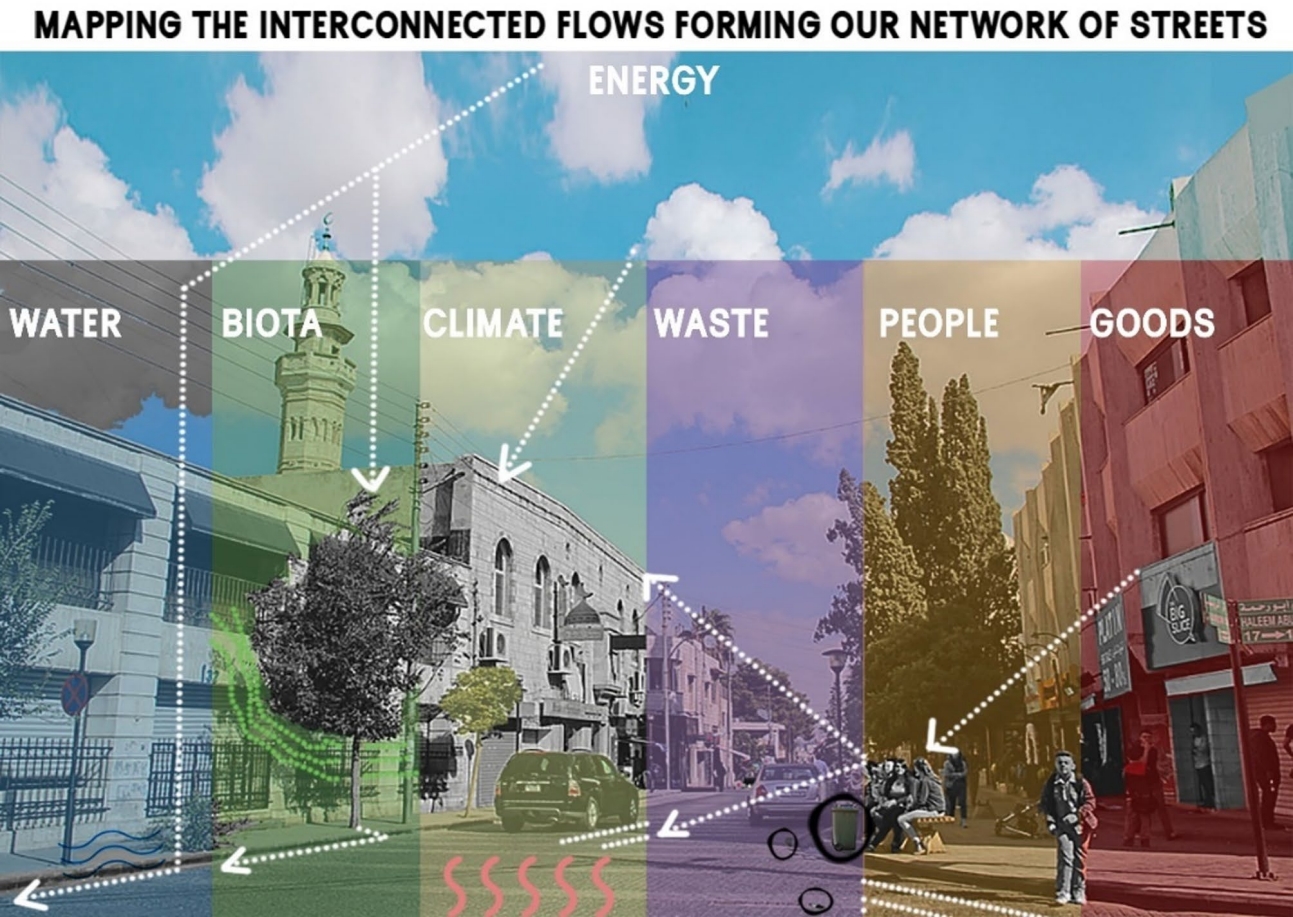
Graphical abstract representation showing intertwined flows within one organ or our ecosystem.

Urban metabolism—a framework conceptualizing cities as living organisms, has emerged as a vital tool for analyzing the flow of resources within urban environments as represented in an abstract form within Fig. 1^1–3^. By examining the inputs and outputs of resources like water, energy, and waste, urban metabolism provides a holistic perspective on city sustainability. This framework has often focused on the broader, metropolitan scale, as seen in foundational studies on cities’ resource cycles and ecological impacts^4^. However, examining urban metabolism at the micro-scale, particularly in street level, offers unique insights into how these dynamics manifest in everyday urban infrastructure. The foundational studies which have established methodologies for assessing cities at metropolitan scales, shed light on such macro-level approaches were often obscure critical neighborhood-level disparities in resource distribution and environmental burdens. Pincetl et al.^6^ argue that aggregated city-scale data homogenizes the lived realities of urban residents, masking inequities in infrastructure quality, access to green spaces, and exposure to environmental risks—a concern acutely relevant to Amman, Jordan, where rapid urbanization has intensified spatial divides between affluent and underserved areas. This study addresses this gap by advancing street-level urban metabolism as a meso-scale analytical framework, focusing on neighborhoods as dynamic units where systemic resource flows intersect with daily human experiences^7^, offering planners granular insights into hyper-local challenges such as unattended green zones, rainwater collection, traffic-induced pollution, and uneven thermal comfort. Streetscapes serve as the conduits for metabolic flows, influencing critical aspects of urban life such as energy use, waste production, water management, and thermal comfort.

The evolution of urban metabolism has been marked by efforts to adapt its principles across scales. Kennedy’s pioneering work formalized city-wide metabolic indicators, while projects like the IABR’s Rotterdam study demonstrated the value of flow mapping for informing urban design. However, as Barles’ analysis of Paris^8^ revealed, most applications remain confined to macro-scale input-output analyses or micro-scale building assessments, neglecting the neighborhood level where infrastructure design directly shapes resource efficiency and equity. Street-level urban metabolism bridges this divide, building on emerging methodologies such as Zhang et al.’s^9^ block-scale energy assessments and Gehl’s^10^ human-centered design principles. Other localized projects, such as North Amsterdam’s Schoonschip, applied urban metabolism principles to neighborhood-scale projects, highlighting their utility for sustainable, resilient design at finer spatial scales^5^. This led to later scholars such as Pestoni explaining different perspectives, emphasizing the adaptability of metabolic analysis across different scales and disciplines^4^.

**Fig. 2:**
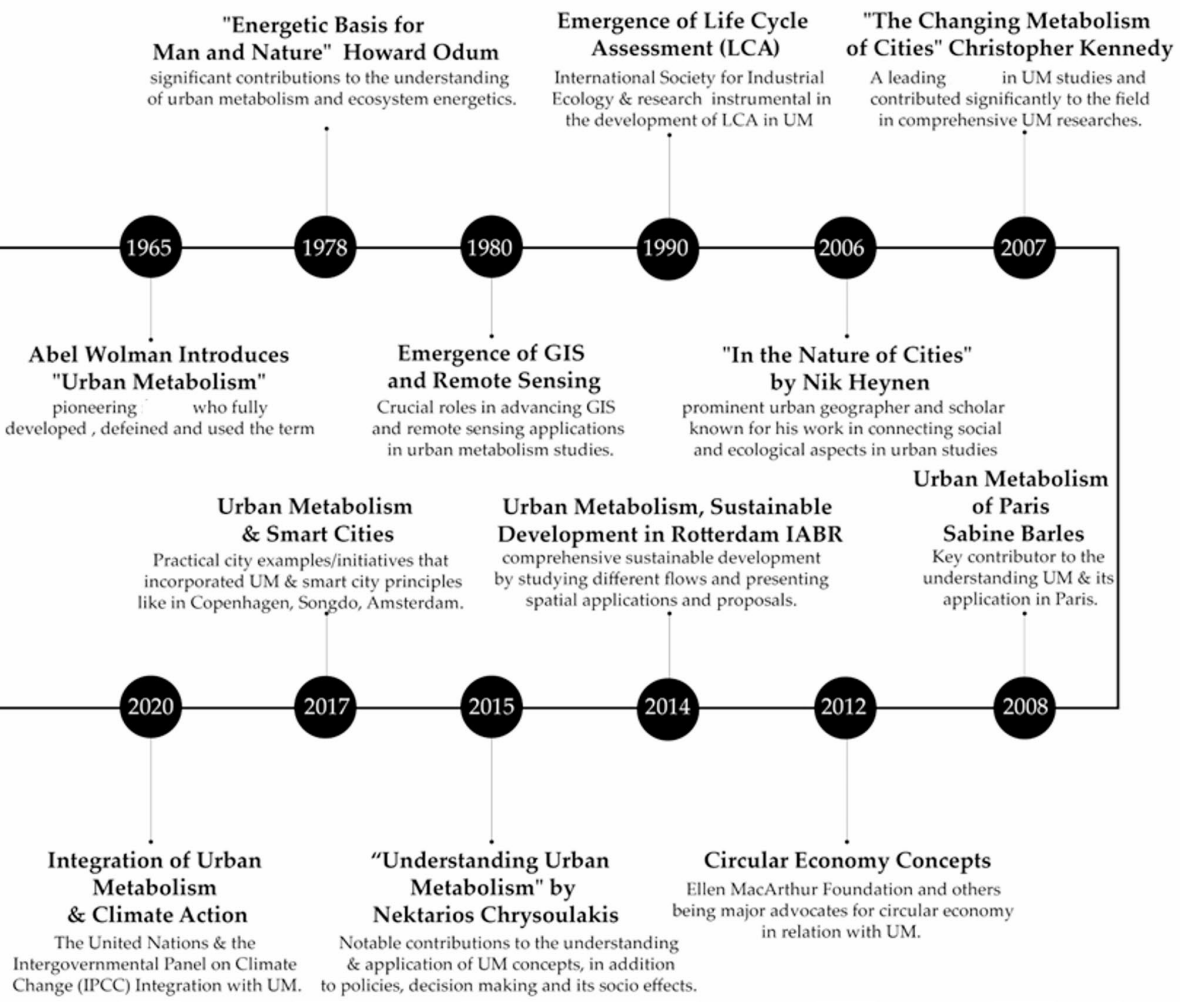
Showcasing urban metabolism timeline, history and its accelerators as a sustainable devolopment tool.

By integrating Kennedy’s indicator framework with the IABR’s flow methodology, this research evaluates seven metabolic processes^11^—biota, climate, goods, people, waste, water, and energy—through spatial metrics, ENVI-met microclimate simulations, and community surveys. This hybrid approach not only quantifies flow efficiencies but also uncovers how urban design choices, such as sidewalk widths or tree canopy coverage, exacerbate or mitigate environmental stressors, especially in high growing cities^12^. This process of metabolic studies has been modified throughout history as seen in Fig. 2, and in recent years, urban metabolism studies integrating methodologies such as flow analysis, material flow analysis (MFA), life cycle assessments (LCA), and comparative data analysis—have proven to be instrumental in guiding urban planning interventions toward sustainability. These frameworks assess critical resource flows, enabling cities to address demands on different flows. For instance, the Amsterdam Circular Strategy employs MFA and LCA to map material flows within the city, creating targeted urban interventions that have effectively increased recycling rates and improved public procurement practices^13–15^. Barcelona provides a similar example, where studies have focused on mapping the city’s material flows as part of its circular economic initiatives, leading to impactful strategies that foster waste reduction and promote local material reuse^15–17^. In Rotterdam, the IABR’s flow analysis applied a multi-faceted urban metabolism approach, producing interventions across multiple urban sectors. These included using geothermal heat to establish new public spaces and repurposing food waste to produce alternatives. Additional innovations involved leveraging sediment deposits to create ecological habitats, such as oyster beds, which support local biodiversity while enhancing CO_2_ sequestration^2,18^. Likewise, in London, Patterson’s urban metabolism framework mapped and assessed resource flows to propose circular economy-centered interventions aimed at reducing resource inefficiencies through advanced waste management and resource reclamation strategies^16,19^. This framework, also discussed in Christopher Kennedy’s work on urban metabolism indicators^3^ offers a scalable methodology for analyzing resource efficiency in cities.

The resultant scoring system provides a replicable tool for diagnosing neighborhood-specific challenges in arid cities, while survey data contextualizes quantitative findings with local perspectives. The study’s significance lies in its dual contribution: methodologically, it advances urban metabolism theory by demonstrating how street-level analysis reveals inequities invisible in city-scale data; practically, it equips planners with strategies to prioritize interventions—from retrofitting flood-prone streets with permeable pavements to expanding shaded pedestrian corridors—where they are most urgently needed. In doing so, the research redefines streets not merely as transport corridors but as vital metabolic organs, where global challenges like climate change manifest in starkly local ways, demanding equally localized solutions.

In Amman—a city grappling with water scarcity, extreme heat, and fragmented green infrastructure—this approach is particularly critical. The study examines four neighborhoods selected for their socio-ecological diversity: Downtown Amman, the historic commercial core; Jabal Amman, a mixed-use tourist hub; the 7th Circle (Sweifeyeh), a rapidly developing commercial zone; and Tla’ Al Ali, a residential area with limited services. These sites collectively represent Amman’s urbanization trajectory, these neighborhoods are historically significant yet recently developed within the past century^5^, underscoring Amman’s rapid urbanization—from a modest town of 2000–3000 residents in the early 1920s to a population of over 2.3 million today forming a metropolis^12^. by Applying these insights at the neighborhood scale can uncover nuanced opportunities for targeted urban planning. Street-level analysis, as this research demonstrates, serves as a promising avenue for creating intervention strategies that enhance sustainability by using urban indicators to enhance urban livability and resilience. Building on urban metabolism research^1,3^.

The aim in short, seeks to explore resource dynamics within urban neighborhoods through a localized metabolic approach, synthesizing Kennedy’s diagnostic indicators with the IABR’s flow-centric methodology. Centered on Amman, Jordan, the investigation evaluates seven interconnected urban systems. By integrating geospatial mapping, ENVI-met microclimate simulations, and community-driven surveys, the research constructs an evaluative framework to uncover neighborhood-specific infrastructural gaps and ecological stressors. The outcomes aim to deliver context-sensitive solutions that promote equitable resource distribution and adaptive capacity in arid cities confronting water scarcity and extreme heat challenges like Amman.

## Methodology

**Fig. 3:**
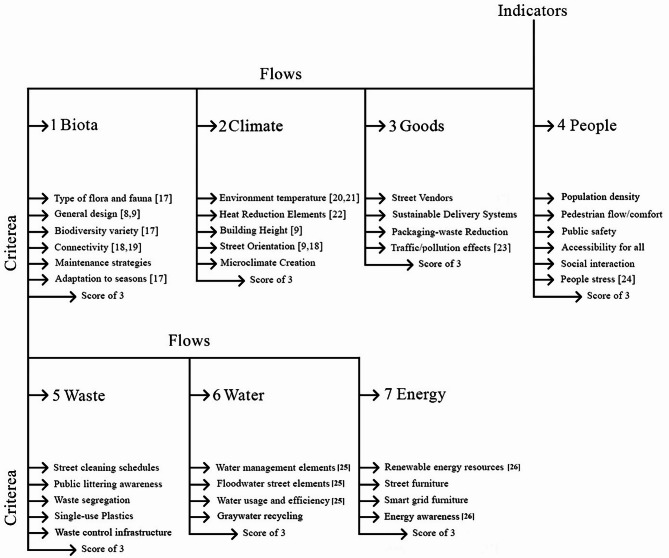
The 7 flows and the criterea assessed as urban metabolic indicators for neighborhoods.

This study employs a street-level urban metabolism framework to evaluate seven interconnected resource flows—biota, climate, goods, people, waste, water, and energy—across four neighborhoods in Amman, Jordan. A 1–3 scoring scale was developed for each flow, with thresholds calibrated to Amman’s arid climate and urban constraints rather than universal benchmarks. For example, biota scoring categorized neighborhoods as “low” (score = 1) if green space per capita fell below 5%, aligning with municipal and WHO guidelines for arid cities, while “high” (score = 3) required over 10% green space, achievable through drought-resistant species like Olea europaea, which can be seen in Fig. 3 and in a more detailed version in appendix A. The final grade for each flow is standardized to a score out of 10, enabling cross-comparative analysis across neighborhoods.

The data collection process included quantitative spatial analysis, surveys, and 3D modeling simulations. Each flow was evaluated based on unique criteria and metrics, with techniques chosen to capture the multifaceted characteristics of each neighborhood’s urban metabolism [Appendix B]. Below, each flow and its criteria are discussed in detail, along with specific data collection and analysis methods.

In assessing biota across the neighborhoods, data collection focused on the diversity of flora and fauna^20^, tree distribution, and green spaces per capita. Key elements of this analysis involved examining the ecological suitability of vegetation types, particularly drought and heat resistance, essential for sustainability in Amman’s arid climate. Observations of general design^11,12^ and connectivity^20,21^ within each area were evaluated.

Climate factors were analyzed through simulations using ENVI-met software, reflecting building heights, vegetation, soil quality, and material types to simulate neighborhood microclimates^23,24^. The study simulated extreme summer conditions using ENVI-met on June 1st at 12 p.m.—Amman’s peak heat period—to evaluate neighborhood thermal resilience. Inputs included precise 3D geometries (building heights, street layouts), surface materials (e.g., asphalt reflectivity), and vegetation data, with meteorological conditions drawn from U.S. Department of Energy EPW files, regionally validated for arid climates. Important climate considerations included heights, orientations, temperature, especially during peak summer months including elements that could provide cooling and sustainable solutions.

For the goods flow, commercial zones were mapped to illustrate their spatial relationship with pedestrian pathways. This analysis included the impact of street vendors, whose presence was examined for environmental effects and potential obstructions. Sustainable practices in local businesses were evaluated by mapping delivery methods and assessing eco-friendly packaging. Traffic congestion was assessed with traffic indices^26^.

The analysis of people’s flow adopted Jan Gehl’s framework, focusing on the interaction of population density, public safety, accessibility, and pedestrian comfort^27^. This involved measuring pedestrian comfort in relation to street congestion and sidewalk availability, as well as evaluating accessibility.

Waste flow analysis included waste management practices and infrastructure supporting cleanliness. This involved reviewing street cleaning schedules, Waste segregation and public awareness of cleanliness were evaluated through survey data, with neighborhoods showing high awareness of recycling and sustainable practices scoring higher.

Water flow was evaluated with a focus on flood impact reduction, water conservation, and recycling practices. Key elements include the use of permeable pavements and rainwater harvesting systems, with neighborhoods showing comprehensive water management strategies earning high scores^28^. Street-level flood management, such as bioswales and rain gardens, was also observed.

Energy flow analysis emphasized renewable energy adoption, energy-efficient infrastructure, and public awareness of sustainable energy practices. The presence of solar panels and efficient lighting systems was noted, with higher scores for areas with active renewable energy use^29^.

Finally, a survey of 320 residents provided critical ground-truthing, linking quantitative metrics to lived experiences. Questions focused on localized perceptions of cleanliness, thermal comfort, and walkability, ensuring scores reflected community-identified priorities, it was distributed through social media platforms to capture resident perceptions across Amman’s neighborhoods. Designed to reflect demographic diversity, the survey achieved a balanced gender distribution and spanned age groups from 18 to 65+, ensuring representation of varied urban experiences. With an aim of approximately 80 forms per neighborhood. This approach ensured each neighborhood’s unique socio-environmental context was proportionally represented while minimizing geographic sampling bias. aiming to reach the methodology prioritized actionable thresholds within Amman’s constraints—for instance, a “high” water score required stormwater strategies feasible under Jordan’s water scarcity policies, even if less ambitious than global standards. Final scores were standardized to a 10-point scale to enable cross-neighborhood comparison, intentionally avoiding cross-regional benchmarks to focus on hyper-local inefficiencies like leaky pipes in Downtown Amman or heat-trapping materials in Tla’a Al Ali. While the approach acknowledges limitations in formal statistical validation (e.g., Cohen’s kappa), its strength lies in synthesizing empirical data, iterative researcher consensus, and community input to a model replicable in arid cities facing similar climate stressors. Details scoring criteria and validation protocols, ensuring transparency in threshold determination and methodological rigor.

## Assessment

Data was gathered through spatial analysis, surveys, and 3D simulations to assess distinct urban flows within each neighborhood. Each flow was examined using the chosen targeted criteria, capturing the unique aspects of each area’s urban metabolism for a comprehensive evaluation which can be visited in appendix B.

### Site A, Downtown, Amman, Jordan

Biota: presents limited and sparse greenery, like Brachychiton populneus, Phoenix dactylifera (date palm), Melia azedarach and Lecokia cretica, Anemone coronaria, alycotome villosa, Sarcopoterium spinosum, Adonis palaestina and Malva parviflora which are common in such altitude of 600 m around the mediterranean even though in very low quantity in the center^20^. leading to insufficient shade and low biodiversity. The green space per capita (7% per 1 km^2^) is below the WHO recommendations, with minimal connectivity between green areas^30,31^.

Climate: Shows high land surface temperatures (36–37 °C in summer) due to low vegetation, predominant concrete surfaces, and lack of passive cooling features such as green roofs or courtyards. After analyzing central downtown Amman with ENVI-met on July 1st at 12 noon, one of the hottest times of year, the median temperature is indicated to be approximately 36 °C. The distance between buildings height and the street scale is moderate, short distance between buildings and sidewalks make people feel more intimate^12^. Buildings with courtyard shapes are influencing temperatures to be reduced by around 1 degree in the analysis, emphasizing on this. There is a lack of other heat reduction strategies, such as green roofs and building materials. many researchers especially of the same region, have studied the passive control methods of traditional courtyard buildings which provide passive cooling. Some monitored a courtyard house within a six centuries and compared it to a modern detached house in ghadames, libya^33^, others by using Envi-met one of the few models solving the interaction between air-plant-buildings to calculate such comfort^34,35^, and analysed how benificial greeneries in addition to courtyards are to passive cooling in egypt^36^, this is an essential element in the jordanian urban fabric, were researches in damascus sharing the same hot arid climate show the great influence of the internal courtyards as a cooling technique^37^.

Goods: Street vendors create pedestrian congestion, impacting flow, cleanliness and even affecting safety of others^39^. Additionally, there is a heavy reliance on single-use plastics, and sustainable delivery systems are limited including hybrid or electric vehicles^40^. There is substantial traffic congestion, estimated by 30 min to travel 10 Km’s in the area, aligning with the highest global congestion aligning with top ranking cities in traffic issues^26^.

People: High population density with more than 1000 buildings per square kilometer, the estimated population of only the residents is over 15,000 people, narrow sidewalks and few seating areas force pedestrians to move quickly, which detracts from the leisurely exploration of the space and social interaction which can be seen in the high stress levels of locals^27^. This is substituted by the plenty of shops which have many awnings and furniture which open between private and public spaces creating interaction zones^12,38^. Public safety and accessibility is also affected by the dense traffic^31^ and inadequate pedestrian infrastructure and ramps.

Waste: While street cleaning is frequent, there is low public awareness of littering in the surveys conducted with only 52% thinking that people are aware and dispose of waste correctly in addition to no waste segregation, and extensive use of single-use plastics. When traced, bins are placed every 15 m, which is highly convenient. In secondary streets, bins are available every 80 m, which also fits the area’s needs^41^.

Energy: Limited renewable energy solutions and energy-efficient street infrastructure contribute to high energy usage. Downtown Amman relies on traditional energy sources, with only 26% coming from renewable sources^42^. The zone is not fully integrated with a smart grid^43^ but projected to be.

When put together in a standardized score this is how each flow performs within site A in Table 1.

**Table 1:** Final standardized score of 10 for all flows in site A.

Flow	Standardized score (out of 10)
Biota	5
Climate	4.67
Goods	3.33
People	5.00
Waste	6.00
Water	3.33
Energy	5

### Site B, Jabal Amman, Amman

Biota: The area features many droughts resistant species, olive trees, Aleppo Pine, Date palm, Quercus Calliprinos (oak), Cupressus Sempervirens (Mediterranean Cyprus) and Pinus Halepensis (Aleppo pine) being native plants^36^, indicating a reasonable level of adaptability to local conditions^20^, green area ratio is the highest between the neighborhoods, with about 7% of green areas within 1 square km, with its lower residence density, it is quite close to the recommendation of the WHO’s minimum green area per individual^30,31^. The main walkable street (Rainbow St) is the core linear passage in the neighborhood while residentials are organized around, there isn’t a wide variety to ensure greenery year-round or native trees that are befitting for sidewalks and pavements like the Mediterranean Hackberry (Celtis australis)^44^.

Climate: ENVI-met analysis conducted on July 1st at 12 noon and at a height of 1.5 m reveals several areas with cooler temperatures, ranging from 33 °C to 34.3 °C. cooler spots align with areas containing substantial vegetation and reduced asphalt, suggesting that less asphalt, narrower streets, and increased greenery contribute to localized cooling. The median temperature is 37.8 °C, building heights range from 6 to 12 m, while the width of streets is around 15 creating intimate short distances^12,38^ Streets mostly run east-west, with buildings facing north-south, providing limited shade. Because of linearity, it still creates a canyon effect that can trap heat and wind in narrow areas.

Goods: Well-organized street vendors enhance social interaction without obstructing pathways. There is heavy reliance on single-use plastics. There is substantial traffic congestion, mixing between tourists, and locals have a high visit rate. Pedestrian accessibility makes it easier for goods transportation.

People: Around 700 buildings per square km are built, making a more relaxed pedestrian environment. Surrounded by 2 M pavements on both sides, walkable streets that prioritize slowing vehicles, while there are 1 to 2 squares they are still lack interaction zones this limits social interaction, particularly for families and the only source of interaction is around shops, cafés and food corners^12,38^.

Waste: Survey results indicate 65% awareness, the highest among surveyed neighborhoods, with 63% of residents unfamiliar with recycling practices. With no initiatives to reduce single use plastics, Adequate infrastructure with 50-liter bins exists every 70–80 m, though public areas with high footfall need more bins.

Energy: Survey shows a good understanding of energy-saving, with only 7% lacking awareness. The walking culture in the neighborhood reduces car dependance, while there are plans for smart grid integration^43^.

When put together with a standardized score this is how each flow performs within site B in Table 2.

**Table 2:** Site B—Final standardized score of 10 for all flows in site B.

Flow	Standardized score (out of 10)
Biota	6.67
Climate	4.67
Goods	5
People	6.11
Waste	6.00
Water	5.00
Energy	5.83

### Site C, Sweifeyeh, 7th circle, Amman

Biota: Green spaces are scarce, covering only 1.5% of the area. The flora consists of drought-tolerant Melia azedarach’s, native Phoenix dactylifera L (date palms), along with some Albizia Julibrissin (Silk trees), these scarce areas are way under recommended percentages^27,28^. Al-Wakalat Street is a good example of a walkable street within the neighborhood with trees lining both sides, serving as an aesthetic yet isolated green feature that is needed all around the neighborhood since it is only 5% of the area.

Climate: after analyzing 100 × 100 m zones which have several empty lots, the median temperature is around 35.39 °C, influenced largely by expansive open areas with vacant sandy lots used as parking spaces which typically register temperatures between 33 °C and 35 °C. This emphasizes an opportunity to create green pockets within these open, unallocated spaces, which can serve as cooling zones and recreational areas and green corridors.

Goods: Street vendors are well-integrated, particularly around commercial hubs. However, sustainable delivery methods and waste reduction measures are absent. High traffic congestion and pollution, particularly during peak hours, was also the biggest problem for users in the survey, The traffic takes more than 30 min per 10 km which impacts air quality and street livability.

People: Densely populated, with 500 buildings per square kilometer. Al-Wakalat Street is pedestrian-friendly, but other areas suffer from narrow sidewalks, poor accessibility for disabled individuals, and obstructive street elements. Commercial areas invite the users and create a ground level interaction zone^38^. But limited public squares or green spaces restrict social gathering options. Heavy traffic causes significant stress among pedestrians and drivers.

Waste: Frequent street cleaning is present, especially in retail areas, but litter remains a problem due to insufficient bins in non-commercial zones. Around 48% think and are part of keeping it clean, the lowest of all three neighborhoods. Waste segregation is lacking, and single-use plastics are prevalent. Garbage bins are clustered in vacant lots, contributing to littering in residential areas.

Water: There are no rainwater harvesting or greywater recycling systems. While the topography naturally mitigates flooding by directing water to lower areas, the area lacks floodwater infrastructure, such as permeable pavements or green spaces to absorb runoff.

Energy: Renewable energy solutions, energy-efficient street furniture, and smart grid integration are absent. Most buildings rely on conventional energy sources, with moderate energy awareness motivated by high electricity costs.

When put together in a standardized score this is how each flow performs within site C in Table 3.

**Table 3:** Final standardized score of 10 for all flows in site C.

Flow	Standardized score (out of 10)
Biota	4.44
Climate	4.67
Goods	4.17
People	6.11
Waste	4.67
Water	4.17
Energy	4.17

### Site D, Tla’ il-Ali, Amman

Biota: The flora includes resilient species like Olea europaea (olive trees), phoenix dactylifera (date palm) mediterranean cypress tree, and Quercus coccifera in addition to some Fraxinus angustifolia. Wild plants on the sidewalks and empty land like Varthemia iphionoides, Tulipa Agenensis and Parentucellia latifolia^20^ are also found, the 4% green area is below WHO standards, limiting biodiversity. The area served as an old cypress tree forest around 5 km away from the center of the neighborhood analysis^45^.

Climate: ENVI-met analysis conducted on July 1st at 12 noon reveals that this area has the highest median temperature at 36.4 °C, with a nearly uniform distribution of temperatures between 36.5 °C and 37.5 °C. Unlike other neighborhoods, Zone D lacks walkable (a lot of asphalt zones), green pockets in empty lots, and courtyard-style buildings, resulting in an absence of natural cooling features. Streets are generally oriented from north to south, meaning most buildings face east and west, which is not ideal for maximizing natural shade or wind flow.

Goods: Street vendors are present but unobtrusive. No sustainable delivery or waste-reducing packaging is in use. Traffic congestion is a significant problem, especially around University Street. This contributes to noise and air pollution, with no visible mitigation strategies.

People: Moderate density with noticeable congestion. The area has 57–60% as a built-up area ranging from mostly residentials to commercials on the main street. Limited walkability due to obstructed paths affects safety and accessibility. These paths create an unwelcoming environment, discouraging walking and forcing people into unsafe conditions repelling users^38^.

Waste: Highest awareness of littering among other zones (68%), though limited infrastructure to support sustainable waste practices [Appendix C], the distance and distribution of bins is distant in mostly unused land as site C. No visible waste segregation methods, and reduction of single use plastics.

Water: Lacks comprehensive water management systems. The area’s altitude helps prevent flooding, but no irrigation systems exist.

Energy: Lacks renewable energy and energy-efficient street furniture. However, there is high resident awareness regarding energy use according to the survey [Appendix C].

When put together in a standardized score this is how each flow performs within site D in Table 4.

**Table 4:** Final standardized score of 10 for all flows in site D.

Flow	Standardized score (out of 10)
Biota	3.98
Climate	4
Goods	4.17
People	5.56
Waste	4.67
Water	4.17
Energy	4.17

**Fig. 4:**
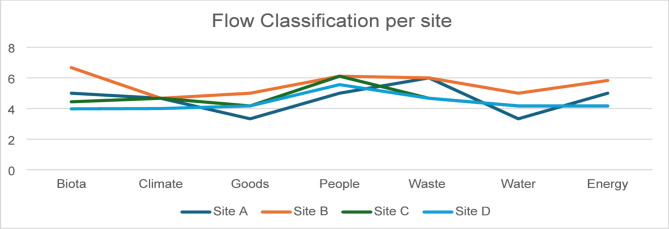
Final illustration of all flows from the previous scored 4 sites.

The combination of all different sites can be seen in Fig. 4 providing the scores in one visual image.

Direct observations: Tourist areas like A & B benefit from a higher allocation of green spaces, this kind of inequality has been assessed in other research like Alexandria in Egypt and Wuhan, China, while a typical occurrence, its consequences are critical^46,47^. Areas with commercial tolls, like A & C have the worst traffic and goods score due to the high traffic congestion, that could affect the people’s pedestrian score also as seen in A. The better waste management sections are more touristic oriented, the low water scores indicate low altitude and bowl-shaped terrain.

### Survey assessment

After collecting 320 forms from the chosen locations. This survey aimed to gather insights into the conditions of urban neighborhoods in Amman, focusing on key aspects of waste management, energy efficiency, and overall street development reflecting the urban metabolism of Amman^3^. The survey can be seen in appendix A. There were eight questions, the first regarding age category, then the place of residence, followed by if the people in the neighborhood are aware of street cleanliness and throw trash correctly, then regarding recycling, and finally energy awareness in the neighborhood, in which issues like cleanliness, recycling and energy can influence sustainable urban development directly^48^. The final 3 questions were more oriented towards checking the results scored before, by asking what are the biggest problems that could be improved in your neighborhood, like green areas, traffic and water management, such factors relate to sustainability indicators directly linked to urban metabolism and tracking biodiversity and green infrastructure as explained in Mori and Christodoulou assessment of urban development^46^, followed by what kind of development would be needed in your neighborhood and finally asking how many steps per day the locals usually take, which gives a glimpse on how walkability is linked to sustainable development and energy reduction^38,49^.

## Results

### Flow assessment results

#### Economic conditions and its impact

Socio economic disparities have largely had a role on physical activity^50^, in wealthier areas like Jabal Amman (B), the higher socio-economic status translates into better infrastructure, resulting in higher biota scores (6.67) and greater walkability, with 44% of respondents walking between 3000–6000 steps daily. In contrast, Tla’ al Ali (D), with lower income levels, struggles with poor green spaces (biota grade 3.98) and pedestrian infrastructure, as 63.89% of respondents walk fewer than 3000 steps daily, relying heavily on cars.

#### Tourism impact

A (Downtown) as the top tourist destination, must balance high footfall from visitors with the needs of residents. This creates unique challenges, such as overcrowding, traffic congestion, and waste management issues. The waste grade (6) is high, suggesting that the municipality prioritizes cleanliness to maintain the area’s attractiveness to tourists^38,39^. While C and D areas are rarely visited by tourists, they tend to have fewer investments, which affects the quality of public spaces. For example, D’s biota grade (3.98) is the lowest, and residents are less likely to walk because the area lacks green, walkable spaces^51^ an understanding effect of urban investment.

#### Urban planning history and planning

Both Downtown Amman and Jabal Amman are older, more established neighborhoods. This means they face legacy planning issues, such as narrow streets, crowded public spaces, and outdated infrastructure. Downtown’s older urban fabric contributes to its congestion and limited green space, as seen in the survey where over 50% of respondents mentioned traffic and 25% called for more greenery. C and D are quite new developments with less visitors leading to improper sidewalks causing poor engagement and safety, this probably is explained more when looking at the functions instead of the date of buildings, since integrated different functions led to more walkability and interaction through “eyes on the street”, an observation stated by the well-known jane Jacobs^52^.

#### Commercial vs. residential focus

All Areas Share a Commercial Focus on Main Roads: Each of these neighborhoods has a mix of commercial and residential zones, with main roads being highly commercial and inner streets more residential. When zooming in detail, A and C have the highest commercial activities, while B and D have the lowest activity, this could explain c’s high traffic congestion problem in the survey^53^, while also explain d’s need for social interacting and zones, in addition to green spaces.

#### Passive cooling solutions induced design

**Fig. 5:**
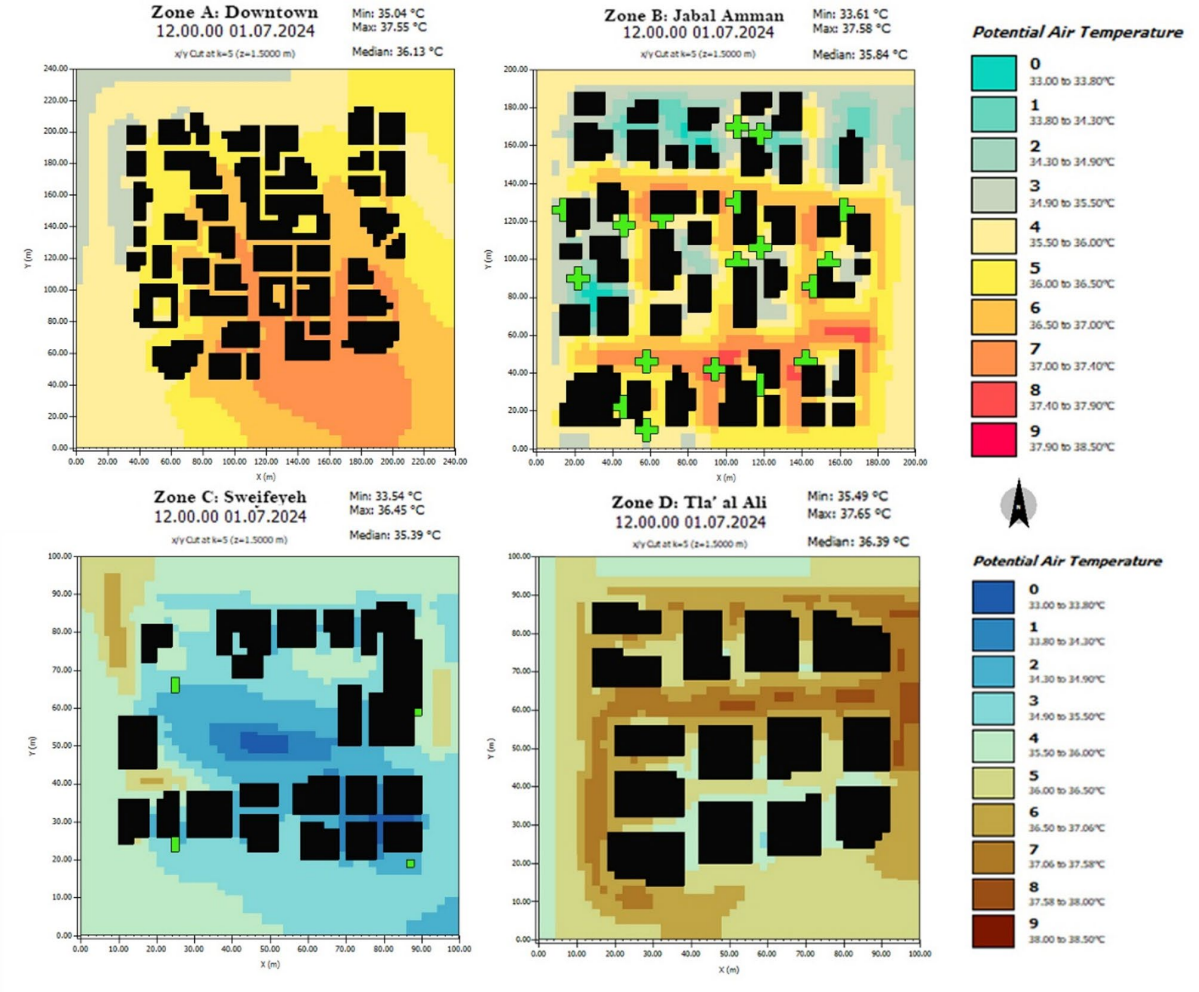
ENVI-met simulations for the blocks within the 4 neighborhoods to evaluate the potential air temperatures.

My analysis of passive cooling solutions in four Amman neighborhoods reveals promising climate-responsive design insights for urban heat mitigation which can be seen in Fig. 5. Conducted on July 1st at noon, using the ENVI-met, the assessment recorded similar median temperatures, ranging from 35.4 °C to 36.4 °C. localized cooling trends emerged in specific configurations. Notably, large unbuilt land masses surrounded by buildings, functioning as quasi-courtyards or superblocks, consistently maintained lower temperatures (33–33.8 °C), even without extensive vegetation. This pattern is particularly evident in Sweifeyeh, where such spaces are informally used for parking, providing a natural cooling effect by limiting asphalt exposure and maximizing shade. Similar cooling benefits are observed in Downtown Amman, where compact courtyard configurations yield temperatures around 35.5–36 °C, cooler than surrounding asphalt-paved streets reaching up to 37.9 °C. Jabal Amman further illustrates the cooling impact of small green zones and non-reflective, pedestrian-focused materials in areas between residential buildings, where temperatures of 33.8–34.3 °C are common compared to asphalt-heavy zones that exceed 37 °C. These findings underscore the potential of integrating superblock-inspired designs, emphasizing green pockets, shaded courtyards, and reduced asphalt to foster passive cooling. Reimagining pedestrian spaces and prioritizing non-reflective, low-heat materials can support a more sustainable, climate-adaptive urban landscape across Amman’s diverse neighborhoods.

### Survey results

**Fig. 6:**
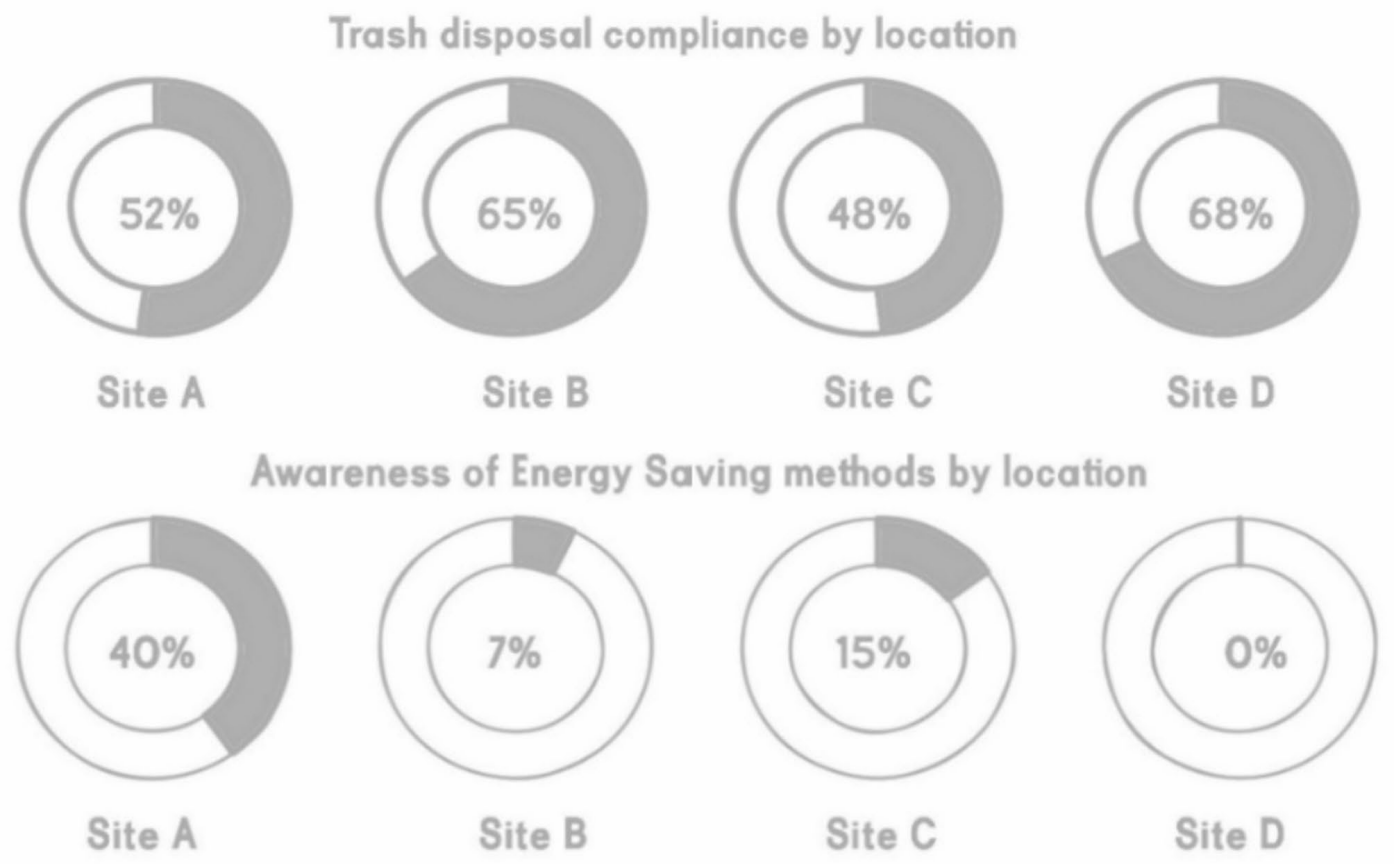
Graphs displaying survey results of the trash disposal and energy awareness through location A–D.

Trash disposal in the survey as seen in Fig. 6 was different from each location to the other despite being in the same city. The highest trash disposal percentage was in “D”, the only unique element was it having the highest residential buildings even though bins in the streets where lower in quantity, indicating people are a bit more aware, which highlights that in Amman, awareness of such conditions can be more significant than having the optimal infrastructure indicating similar points to Gao’s community awareness indicators^54^, location A and C have the highest amount of commercial and retail shops, while B with its high amount of shops still has a good ratio, could be because it’s a generally wealthier area, linking socio-economic structure of better income levels with better waste management practices^55^.

**Fig. 7:**
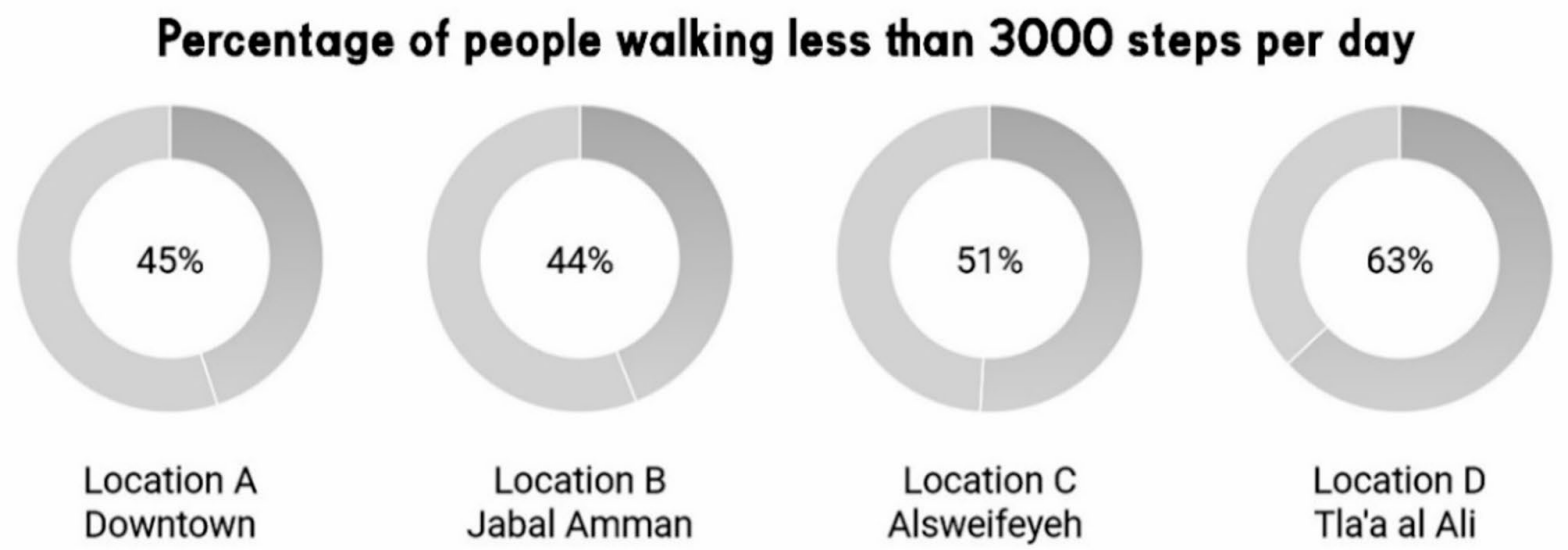
Graphs displaying survey results of the steps walked per day through locations A–D.

#### Correlation between walkability and green spaces

**Fig. 8:**
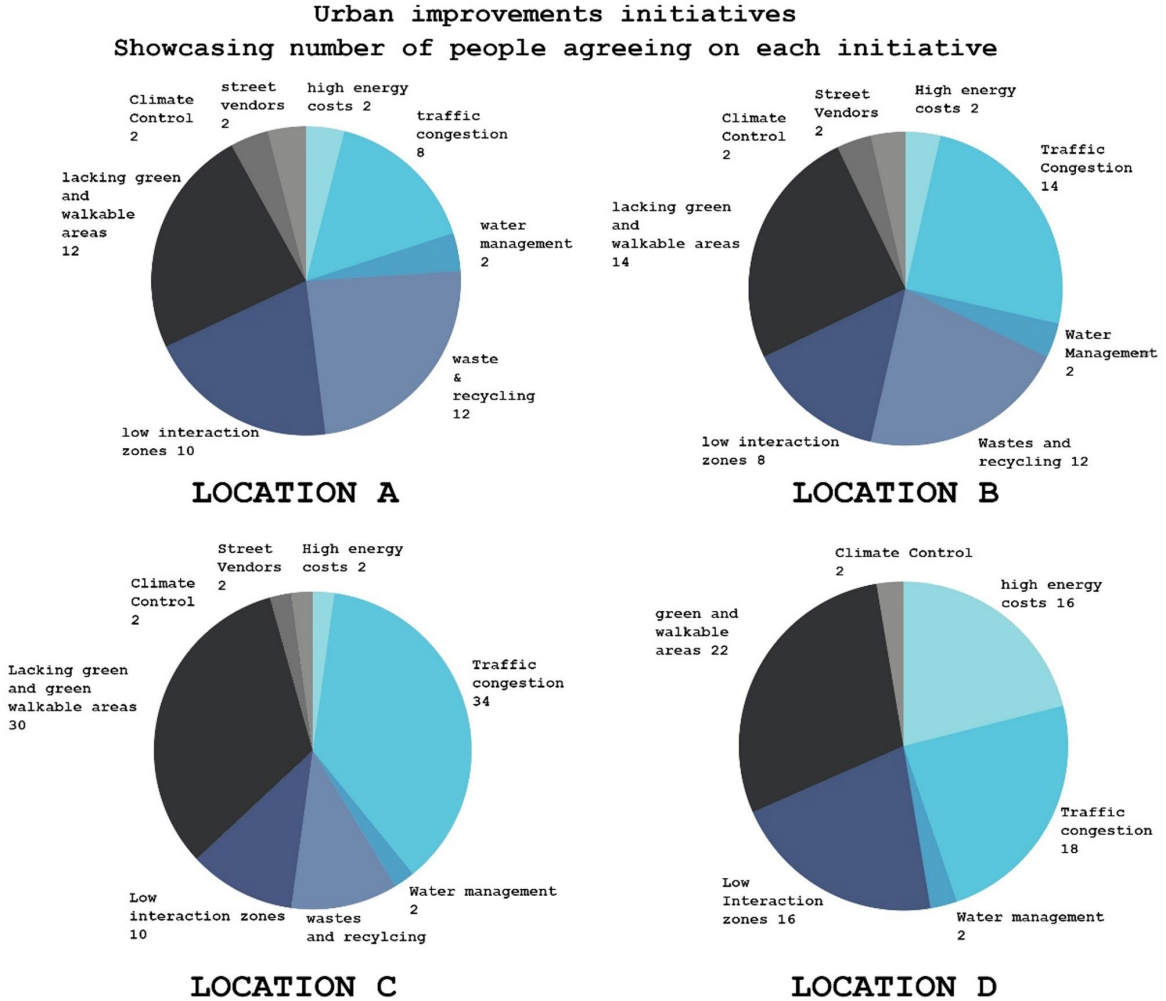
Urban improvement initiatives showcasing survey results from location A–D.

Jabal Amman (B) with a higher biota grade (6.67), and a people flow of 6.11 has the highest in real time amount of greenery and pedestrian comfort, while Sweifeyeh (C) has a lower biota score. The relative lack of green spaces likely contributes to lower pedestrian comfort which effectively can be seen in the survey. 63.89% of respondents in D reported walking fewer than 3000 steps per day being the lowest of all neighborhoods as noticed in Fig. 7. Therefor residents heavily rely on cars, as indicated by survey responses in Fig. 8 showing dissatisfaction with walking infrastructure, whereas with B where participants reflected pedestrian comfort, emphasizing increased physical activity linkage to green spaces^56^. In location D, the lowest biota grade was found, from the least amount of green infrastructure, as confirmed by both the survey and the analysis. This hints for more development needs, 19.44% of respondents identified the need to increase greenery and add shaded areas, which closely aligns with the low biota grade (3.98) from the analysis, the need for better green infrastructure development to enhance such issues is clear in Ewing and Handy’s built environment highlights^57^.

#### Initiatives insights

**Fig. 9:**
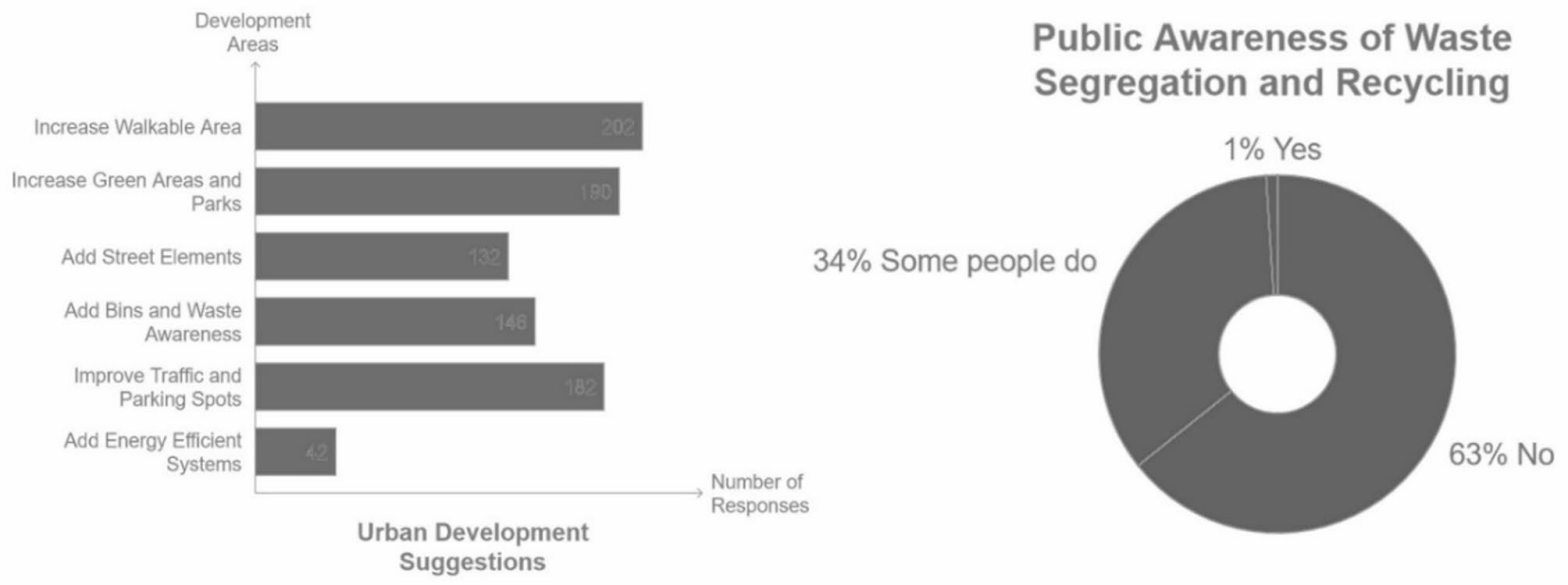
Urban development suggestions and people’s awareness regarding recycling answers from A–D.

Neighborhoods like Tla’ al Ali (D) and Sweifeyeh (C), which received lower grades in the biota analysis, are viewed by survey participants as lacking in greenery, with residents calling for improvements in parks, trees, and shaded areas where around 26% of C’s residents asked for increasing walkable areas and over 28% asked for solving traffic congestion regarding urban development suggestions seen in Fig. 9. Conversely, Jabal Amman (B), with a higher biota grade, showed fewer complaints about greenery, confirming that neighborhoods with lower biota scores are perceived as having insufficient greenery by the people in the survey.

### Correlation analysis

This analysis builds on the early statistical contributions of Ronald Fisher^58^ to explore the relationship between urban flows (biota, climate, waste, etc.) and residents’ perceptions using correlation analysis to measure the strength of relationships between subjective perceptions (from the survey) and the flow grades, by using Pearson’s correlation coefficient (*r*), with statistical significance assessed through two-tailed *p*-values and 95% confidence intervals (CI). Key findings include:

- Biota and walkability: Strong positive correlation (*r* = 0.85, *p* < 0.001, 95% CI [0.79, 0.89]) between biota / green spaces and perception of walkability across all sites. Jabal Amman, with a biota grade of 6.67, had the highest walkability ratings in the survey, where 44% of respondents walked between 3000 and 6000 steps per day.
- Waste management and cleanliness perception: Moderate positive Correlation (*r* = 0.78, *p* = 0.013, 95% CI [0.66, 0.86]). between waste management grades and cleanliness satisfaction. Areas like Downtown and Jabal Amman, with high waste management grades of 6, showed 60–65% satisfaction with waste management in the survey.
- Goods and traffic congestion: Strong negative correlation (*r* = − 0.73, *p* < 0.001, 95% CI [− 0.82, − 0.61]). between goods grades (street vendor activity, traffic) and pedestrian comfort. The negative correlation reflects how congestion and overcrowding commercial spaces lead to lower pedestrian comfort. Seen in A. Downtown, with a goods grade of 3.33, over 50% of respondents highlight traffic as a major concern.

A multivariate linear regression model [Appendix D] was applied, controlling for socio-economic factors. Results confirmed:

- Biota is the strongest predictor of walkability (β = 0.69, *p* < 0.001).
- Goods flow (traffic congestion) significantly reduced pedestrian comfort (β = − 0.58, *p* = 0.007).

This analysis demonstrates robust statistical linkages between objective metabolic performance and subjective resident experiences, addressing the reviewer’s concerns about methodological transparency.

## Discussions

### Theoretical and literature review contribution in theory and practice

The study’s observations on neighborhood-level provide deeper connection with the people and built environment on the contrast to larger scale studies with large scale results, variations in green infrastructure and walkability, for example, reveal how the distribution of greenery can enhance thermal comfort and air quality. This aligns with literature, that green infrastructure is crucial for moderating microclimate effects and enhancing overall resilience^1^. In denser neighborhoods, limited green space and high traffic densities correlate with increased temperatures, reinforcing studies that link built-up areas and a lack of vegetation to urban heat island effects^59^, additionally the zones with less traffic which is in an alarming rate throughout the surveys and the analysis, effectively boost urban livability. This affirms calls within urban planning literature for strategies that prioritize pedestrian accessibility and integrate green spaces into high-traffic urban areas to enhance quality of life^12^.

Certain socio-economic factors—like the availability of resources for green infrastructure and waste management—can significantly impact residents’ perceptions of environmental quality. This finding aligns with studies suggesting that socio-economic disparities can amplify or mitigate urban environmental issues^60,61^, indicating a need for localized planning policies that respond to both physical and socio-economic contexts. The results suggest that integrating resident perceptions into the analysis of urban flows adds a critical dimension to understanding how urban metabolism influences livability, which is often overlooked in conventional metabolism studies, highlighting the importance of incorporating subjective experiences alongside quantitative data in assessments^62^.

Zone B, and some locations in C, offer empty areas with trees, greenery and in the case of B walkable streets with more biodiversity, showing cooler street temperatures and improved outdoor comfort. This observation not only contributes to the urban metabolism literature by showcasing the effects of greenery on microclimate control but also underscores the originality of applying these insights at a micro-scale in the Middle East, where studies on temperature modulation at this scale remain limited.

### Originality and relevance of findings

The originality of this lies in its integration of neighborhood-specific urban metabolism analysis with dents’ feedback and environmental metrics such as temperature fluctuations influenced by spatial planning choices. Unlike broader-scale studies, this research examines how urban temperature, green infrastructure distribution, density, and surface materials, contribute to the metabolism of cities.

This type of neighborhood-level, microclimate-focused analysis has not been widely applied in the Middle East and hasn’t been conducted in Amman. By examining temperature variation and urban metabolism at the street level in an arid city like Amman, this study contributes original insights into urban literature, addressing a significant gap and providing a model for similar urban environments facing extreme heat. The findings align with recent calls for urban studies to incorporate environmental dynamics at a granular scale^59^, offering a blueprint for localized, adaptive urban strategies that resonate with residents’ lived experiences and the distinct climate.

### Applications and implications in urban planning as feasible solutions from the study

Biophilic pocket parks and temperature moderation: Amman has many empty land parcels, which could be converted into biophilic pocket parks. This solution, derived from analyzing waste bin locations and aerial photos of void spaces, proposes integrating natural elements into neighborhoods to foster connections between residents and nature. Creating biodiverse corridors and transforming underutilized plots into pocket parks or community gardens can enhance biodiversity while providing recreational spaces, as highlighted in survey feedback. Preliminary findings suggest that adding green pockets between blocks in sizes like 30 by 30 or 50 by 50 square meters could reduce local temperatures by around 2–3 °C. This demonstrates the potential for green spaces to mitigate urban heat, offering both environmental and health benefits.

Nature-based urban drainage solutions: The city’s water management score is critically low. Implementing green infrastructure, such as bioswales and rain gardens, particularly in densely populated areas like Downtown Amman, could improve stormwater management and the aesthetic quality of streetscapes. Such solutions address both flood risks and the residents’ expressed desire for more green spaces.

Pedestrian-centric urbanism and microclimate creation: By recreating successful pedestrian-prioritized zones, such as those seen in Rainbow Street, a network of pedestrian-friendly areas can be developed in different neighborhoods. Linking commercial hubs with residential zones would create safer, walkable environments that support local businesses and enhance walkability city-wide. In addition, pedestrian-centric areas with shaded trees on both sides of the walkable streets, as observed in Rainbow Street, contribute to a cooler microclimate and reduced temperatures, further encouraging pedestrian comfort, this can also be managed extend existing shaded walkways by 500 m using drought-resistant vines (Vitis vinifera) on pergolas, prioritizing connectivity to the Darat al Funun art gallery. Finally, a smart addition to Site C, would be to retrofit Al-Wakalat Street’s asphalt with high-albedo concrete (reflectivity ≥ 0.5) to reduce surface temperatures by 3–5 °C.

Waste management can drastically see improvements in different methods by exploiting the high waste in the commercial neighborhoods, with real-time fill-level sensors along zone A’s King Hussein Street, prioritizing high-footfall zones can be an efficient solution. This can also be funded via a “Clean tourism tax” (1% surcharge on visitors) especially in tourist zones in a weak economic region. When it comes to waste from street vendors, agreements with street vendors to adopt compostable packaging can be enforced through municipal licensing renewals as a solution.

Courtyard building and superblock structures: To address microclimate and biodiversity challenges, especially in Amman’s arid climate, adopting courtyard-style buildings and localized adaptations of the Barcelona superblock model can reduce heat and create internal microclimates. Initial observations suggest that courtyards and open spaces between buildings can reduce average temperatures by 2–3°C. Expanding such designs across Amman could not only enhance cultural identity but also contribute to a more sustainable urban environment. Superblocks and passive cooling are not abstract ideals but actionable extensions of Amman’s existing urban DNA. This approach would help develop local economic hubs while supporting biodiversity and addressing high urban temperatures through localized green infrastructure^65^. Amman’s organic urban fabric, with its compact, pedestrian-oriented historic cores (e.g., Jabal Amman), already embodies principles of the Barcelona superblock model. Retrofitting newer areas like Sweifeyeh into car-restricted zones with internalized traffic (e.g., limiting through-traffic to peripheral arteries) is achievable, as evidenced by this study’s ENVI-met simulations showing that quasi-courtyard configurations reduce temperatures by 2–3°C. When subjected to the existing urban planning policies, Jordan’s national urban policy^63^ emphasizes “human-scale neighborhoods,” which would be aided by creating a governance framework to pilot superblocks in low-congestion zones like Tla’ Al Ali or revisiting one of the most built typologies in the region (70).

## Conclusions

### Summary key findings

Commercial congestion vs. walkability: The inverse relationship between commercial congestion and pedestrian comfort. In highly commercialized areas like Downtown Amman, despite their economic vibrancy, the traffic congestion and overcrowded streets negatively impact walkability. This highlights a need for balanced zoning that reforms to balance economic activity and walkability which links cities with a higher ratio of green spaces to commercial density perform better in terms of livability^66^.

Perceptions of waste management: There is a strong link between waste management efficiency and residents’ perceptions of cleanliness. While waste management flow in Tla’ al Ali scored lower, residents there did not rate cleanliness as poorly, revealing a disconnect between actual urban metabolic performance and subjective perceptions. This suggests that residents’ experiences may not always align with quantitative flow data. For instance, wealthier areas had more bins and cleaner atmospheres, yet residents still ranked the area as needing more cleanliness. This finding underscores the importance of accounting for socio-economic dynamics in metabolism assessments^67,68^.

Impact of Socio-Economic factors on urban resources: Affluent areas (e.g., Jabal Amman) exhibit superior biota (6.67/10) and energy performance (5.83/10), underscoring the need for targeted investments in underserved neighborhoods. Wealthier and tourist-attracting areas, such as Jabal Amman, benefit from better infrastructure, energy efficiency, and green spaces, which contribute to higher walkability and sustainability.

Biota and walkability correlation: Strong correlation (*r* = 0.85, *p* < 0.001) confirms green spaces enhance walkability; modest interventions (e.g., pocket parks) yield disproportional benefits^69^.

### Regional contexts

This study’s findings resonate with broader MENA urban challenges while underscoring Amman’s unique context. Like Elagib & Al-Saidi’s (2020) analysis of Cairo and Riyadh^70^, Amman faces acute water scarcity exacerbated by aging infrastructure and population growth. However, our street-level approach reveals hyper-local disparities: Downtown’s water score (3.33/10) reflects century-old pipes under tourist-heavy streets, whereas Tla’ Al Ali’s newer residential zones (4.17/10) benefit from modernized networks—a granularity absent in regional-scale studies.

Similarly, while Al Khaled et al.^71^ demonstrate Gulf cities’ reliance on energy-intensive cooling, Amman’s fiscal constraints necessitate passive, low-tech strategies. Our ENVI-met results show that courtyard retrofits and land pockets reduce peak temperatures by 1–2 °C, outperforming Dubai’s misting systems in cost-effectiveness if not magnitude. This divergence highlights how urban metabolism frameworks must adapt to local economic realities, particularly in non-oil-dependent MENA cities. These comparisons affirm that while regional climatic and infrastructural patterns exist, neighborhood-scale interventions require context-specific calibration—a gap this study fills by linking metabolic flows to Amman’s socio-ecological fabric.

### Limitations

While these neighborhoods provide a strong representation of different urban conditions within the city, they may not fully capture the complexity of Amman’s broader urban metabolism. Expanding the study to include additional districts would strengthen the generalizability of the findings. Even though the neighborhoods represent standard blocks of the city. Finally, some metabolic flows, such as energy and water efficiency at the building level, were assessed based on broader indicators rather than direct real-time monitoring. While this aligns with existing urban metabolism methodologies, incorporating more precise tools, such as smart meters, remote sensing, helps to provide a more granular understanding of resource distribution and efficiency. This can also be noticed in city-wide factors—such as regional transportation networks, industrial activity, and cross-district resource flows—also influence urban metabolism. Future research could bridge these scales by linking micro-scale findings to macro-scale city planning and infrastructure strategies.

## Electronic supplementary material

Below is the link to the electronic supplementary material.

Supplementary Material 1

## Data Availability

All data generated and analyzed during this study are included in this published article and its supplementary information files.

## Declarations

### Identifiable individuals statement

This study does not include any identifiable information or images of human participants.
