# Supplementary material for: Street level urban metabolism as a tool for mapping urban flows in Amman’s neighborhoods
Source: Sci Rep. 2025 May 28;15:18654. doi: 10.1038/s41598-025-03821-y (PMC12120028; doi:10.1038/s41598-025-03821-y)
Supplement: Supplementary file 1 — Supplementary Material 1 [file 41598_2025_3821_MOESM1_ESM.docx]

**Street level urban metabolism as a tool for mapping urban flows in Amman’s neighborhoods.**

Supplementary Data & Appendixes:

Appendix A, Methodology indicators table:

| Flow | Criteria | Assessment Technique  (Score out of 3) | Score 1 | Score 2 | Score 3 |
| --- | --- | --- | --- | --- | --- |
| Biota [2] | Type of flora and fauna [17] | Ecological evaluation regarding adaptability | Minimal drought / heat resistance | Moderate drought or heat resistance | High drought or heat resistance |
|  | General design [8,9] | Design based analysis | Basic design, limited | Functional design, moderate | Innovative design, highly effective |
|  | biodiversity variety [17] | Ecological analysis and percentage/ratio evaluation | Low biodiversity ratio | Moderate biodiversity ratio | High biodiversity ratio |
|  | Connectivity [18,19] | Ecological analysis regarding green corridors | Limited connectivity | Some connectivity, but with gaps | Extensive connectivity |
|  | Maintenance strategies | Efficiency and sustainability assessment | Maintenance is inefficient | Maintenance is somewhat efficient | Low maintenance, highly efficient |
|  | Adaptation to seasons [17] | Seasonal planting strategies and invasive species | Poor adaptation | Moderate adaptation | Excellent adaptation |
| Climate  [2] | environment temperature [20,21] | Measurement of street and surroundings temperature and reflective elements | High temperatures, low comfort | Moderate temperatures, some discomfort | Low temperatures, comfortable for pedestrians |
|  | Heat Reduction Elements [22] | Assessment | Few or no Heat Reduction | Some heat reduction elements present | Abundant heat reduction |
|  | Building Height [9] | Analysis based on Jan Gehl analysis. | Buildings height poor shading | Mixed heights, some shading | Optimal shading element |
|  | Street Orientation [18] | Sun exposure analysis | Poor orientation, excessive sun | Moderately oriented or optimized | Excellent orientation/ shade |
|  | Microclimate Creation | Assessment | Minimal efforts | Moderate modification | Comprehensive modification |
| Goods [2] | Street Vendors | Environmental impact and obstruction design analysis | High disruption, poor hygiene | Moderate or some disruption | Minimal waste, well-managed |
|  | Sustainable Delivery Systems | Use of bike couriers, electric vehicles assessment | Predominantly fossil fuel vehicles, high | Mix of fossil fuel and some sustainable vehicles | Predominantly sustainable vehicles, low |
|  | packaging-waste | Reduction initiatives assessment | High levels of single use, no reduction | Some initiatives, partial reduction | Comprehensive eco-friendly packaging |
|  | Traffic/pollution effects [23] | Traffic and pollution effects analysis | Significant congestion | Some strategies mild congestion | Well-plan, minimal congestion |
| People  [2] | Population density | Density impact on street congestion and usability | High density and overcrowdness | Moderate manageable density | Low density with optimal street use |
|  | Pedestrian flow/comfort | Design based analysis | Narrow sidewalks, obstructed | Adequate sidewalks, some seating areas | Wide sidewalks, no obstruction |
|  | Public safety | Lighting, visibility, security | Poor lighting, visibility, security | Adequate lighting, moderate visibility | Excellent lighting, visibility, security |
|  | Accessibility for all | Ramps, tactile paving analysis | Limited or no accessibility | Some accessibility features present | Comprehensive accessibility |
|  | Social interaction | Evaluating social interaction | Few or no social interaction spaces | Some social interaction spaces | well-designed social spaces |
|  | People stress [24] | Gallup world ranking for emotions | no greenery, traffic & stress levels | Moderate greenery, stress levels | low stress related factors |
| Waste  [2] | Street cleaning schedules | Schedule and frequency evaluated | Inconsistent schedules | Regular but infrequent cleaning | well-coordinated schedules |
|  | Public littering awareness | Survey | Low awareness, high littering | Moderate awareness/ littering | High awareness, minimal littering |
|  | Waste segregation | Survey | Limited segregation | Some segregation systems in place | Comprehensive segregation |
|  | single-use plastics | nearby shops evaluated | High usage of single-use plastics | Moderate reduction efforts | Significant reduction efforts, |
|  | Waste control infrastructure | Design analysis | Few bin points, poorly maintained | Close to adequate number of bins | Adequate bins and well-maintained |
| Water  [2] | Water Management Elements [25] | permeable pavements, rainwater harvesting, drought combating design | Predominantly impermeable surfaces | Partial implementation of permeable surfaces | Extensive use of permeable well-integrated systems |
|  | Floodwater Street Elements [25] | Bioswales, rain gardens design analysis | Absent or minimal floodwater management | Presence of some bioswales, rain gardens | Comprehensive floodwater management |
|  | Water Usage and Efficiency [25] | water conservation methods in the city | Predominant use of traditional irrigation | Partial adoption of water-efficient systems | Extensive use of efficient water systems |
|  | Greywater Recycling | Design analysis | No greywater recycling systems | Limited greywater recycling initiatives | Comprehensive recycling systems |
| Energy  [2] | Renewable energy sources [26] | Efficient street lighting, solar panels, public transportation energy usage | Traditional lighting, high energy consumption | Partial use of energy-efficient lighting | Comprehensive use of energy-efficient lighting |
|  | Street furniture | Assessing Benches, kiosks solar powered etc. | No energy-efficient street furniture | Some energy-efficient furniture | Widespread use of efficient furniture |
|  | Smart grid integration | Assessing smart grid integration in the city | No smart grid integration | Partial smart grid integration | smart grid real-time monitoring |
|  | Energy awareness [26] | Survey | No awareness | Basic energy consumption | Comprehensive energy awareness |

Table S1. Introducing the flows to be analyzed and the assessment technique followed.

Appendix B, collected data and graphic visuals presented used for site analysis assessment:

Site A, Downtown, Amman, Jordan.

Assortment of street perspectives in figure S1 showcasing the overall atmosphere of the Site. Figure S1. marked images around downtown area presenting the overall atmosphere refferenced in figure S2. (Photographs A, B, D and F taken by the author (Anas Tuffaha) in 2023, Photographs C and E obtained from Google Street View (© Google, 2023), accessed via Google Maps in 2023, available at https://www.google.com/maps/. Imagery was used for academic, non-commercial purposes in accordance with Google’s Terms of Service.)

Flow 1: Biota Flow analysis presented in Table S2:

| Biota | Type of Flora and Fauna | General Design | Urban Biodiversity | Connectivity | Maintenance & Irrigation | Adaptation to Seasons |
| --- | --- | --- | --- | --- | --- | --- |
| Final Score | 2 | 1 | 2 | 1 | 1 | 2 |

Table S2. Site A – Biota flow analysis.

Figure S2. Downtown sample google earth aerial photo, followed by the green areas presented in green (satellite imagery obtained and generated by the author (Anas Tuffaha) using Google Earth Pro (Version 7.3.6.9345, © Google, 2023), available at https://www.google.com/earth/. Imagery was edited for academic visualization purposes using Adobe Photoshop CC 2015 (Version 16.0, © Adobe Inc., https://www.adobe.com/). Used in accordance with Google’s permissions guidelines (http://www.google.com/permissions/geoguidelines/attr-guide.html).)

The area includes species such as Brachychiton populneus, Phoenix dactylifera (date palm), and Melia azedarach, Lecokia cretica, Anemone coronaria, Alycotome villosa, Sarcopoterium spinosum, Adonis palaestina, and Malva parviflora. while the green area per 1 km² is 7% as seen in figure S2. Connectivity between green spaces is minimal, and there are no green corridors. Maintenance is conducted weekly by municipal workers, but sustainable irrigation systems are absent, with basic tree basins that do not support natural irrigation.

Flow 2: Climate Flow analysis presented in Table S3:

| Climate | Existing temperature of the street | Heat Reduction Elements | Building Height | Street Orientation by Maximizing shade and wind flow | Microclimate Creation, through design modification |
| --- | --- | --- | --- | --- | --- |
| Final Score | 1 | 1 | 2 | 2 | 1 |

Table S3. Site A – Climate flow analysis

Figure S3. ENVI-met analysis for potential air temperature on 12:00 , 1^st^ of July 24 at 1.5 meter section in zone A.

Evidence of high land surface temperatures (LST), particularly in summer, reaching 30-40°C. The lack of vegetation exacerbates heat retention, with an observed increase in LST of around 5% in 2020, despite some mitigating effects of density and built-up areas [29]. ENVI-met analysis of downtown Amman on July 1st at 12 noon indicated in figure 12 with a median temperature of 36°C, with most areas maintaining temperatures between 36-37°C and courtyards offering slight cooling relief at around 35.5°C. Buildings are typically 3-4 floors high, create moderate shading in the morning and late afternoon, but with low vegetation. As presented in figure 13, The street orientation is mostly northeast and southeast oriented.

Figure S4. The sample area presented in different hours of the day, showcasing shade,heights, and exposure. (3D models were created by the author (Anas Tuffaha) using SketchUp Make 2017 (© Trimble Inc., Version 17.2.2555, https://www.sketchup.com/) and subsequently edited using Adobe Photoshop CC 2015 (© Adobe Inc., Version 16.0, https://www.adobe.com/). All figures were created by the author and are original content.)

Flow 3: Goods Flow analysis presented in Table S4:

| Goods | Street Vendors | Sustainable Delivery Systems | Packaging Waste Reduction | Traffic and pollution effects |
| --- | --- | --- | --- | --- |
| Final Score | 1 | 1 | 1 | 1 |

Table S4. Site A – Goods flow analysis

Street vendors obstruct pedestrian flow, reducing available walking space to one or two meters [36]. Sustainable delivery systems, such as electric vehicles, are limited. Single-use plastic packaging is heavily relied upon, Goods transportation exacerbates severe traffic congestion, with an estimated 30-minute travel time for 10 kilometers showcased in figure S5 respectively.

Figure S5. A google maps traffic illustration on the left showing 30 min per 10km, followed by how frequent people cut the road in different areas showing their organic paths taken explaining routine and denisty. (3D models were created by the author (Anas Tuffaha) using SketchUp Make 2017 (© Trimble Inc., Version 17.2.2555, https://www.sketchup.com/) and subsequently edited using Adobe Photoshop CC 2015 (© Adobe Inc., Version 16.0, https://www.adobe.com/). All figures were created by the author and are original content.)

Flow 4: People Flow analysis presented in Table S5:

| People | Population density and its impact | Pedestrian flow and comfort | Public safety | Accessibility for all | Social interaction spaces | people stress |
| --- | --- | --- | --- | --- | --- | --- |
| Final Score | 2 | 2 | 2 | 1 | 2 | 2 |

Table S5. Site A – People flow analysis

Figure S6. pedestrian obstructions and elements (Image obtained from Google Street View (© Google, 2023), accessed via Google Maps in 2023, available at https://www.google.com/maps/. Imagery was used for academic, non-commercial purposes in accordance with Google’s Terms of Service.)

The area is marked by high population density, with over 1,000 buildings per square kilometer and more than 15,000 residents. Narrow sidewalks, typically one to two meters wide, and minimal seating options limit pedestrian comfort. Social interaction is supported by street vendors, cultural activities, and the architectural scale, where 15-meter-high buildings as presented in figure S7 from a 3d point of view and consistent street widths foster interaction between street-level activities and building occupants. However, stress levels are high, with 42% of the population in Jordan reporting stress the previous day [24]. Accessibility is uneven, with ramps available on main streets but inconsistent in secondary streets, and pavements are incomplete.

Figure S7. people and building relationships with people density on streets marked in orange dots on the right. (3D models were created by the author (Anas Tuffaha) using SketchUp Make 2017 (© Trimble Inc., Version 17.2.2555, https://www.sketchup.com/) and subsequently edited using Adobe Photoshop CC 2015 (© Adobe Inc., Version 16.0, https://www.adobe.com/). All figures were created by the author and are original content.)

Flow 5: Waste flow analysis presented in S6:

| Wastes | Street Cleaning and maintenance Schedules | Public Awareness towards littering | Waste segregation systems | Reduction of single use plastics | Waste management infrastructure design |
| --- | --- | --- | --- | --- | --- |
| Final Score | 3 | 1 | 1 | 1 | 3 |

Table S6. Site A – Waste flow analysis

Figure S8. presenting the public bins and residential area bins on the map. (satellite imagery obtained and generated by the author (Anas Tuffaha) using Google Earth Pro (Version 7.3.6.9345, © Google, 2023), available at https://www.google.com/earth/. Imagery was edited for academic visualization purposes using Adobe Photoshop CC 2015 (Version 16.0, © Adobe Inc., https://www.adobe.com/). Used in accordance with Google’s permissions guidelines (http://www.google.com/permissions/geoguidelines/attr-guide.html).)

The area benefits from frequent street cleaning schedules, with daily waste collection and precise timing. However, public awareness of littering is low as included in the survey. Waste segregation systems are absent, and the widespread use of single-use plastics persists, despite these challenges, public bins are placed every 15 M on the main commercial street and every 80 M on secondary streets as in figure S8 graphically presented.

Flow 6: Water flow analysis presented in Table S7:

| Water | Water management elements | Floodwater street elements | Water usage and efficiency | Greywater recycling systems |
| --- | --- | --- | --- | --- |
| Final Score | 1 | 1 | 1 | 1 |

Table S7. Site A – Water flow analysis

The area has minimal water management elements, with impermeable surfaces dominating and limited use of permeable pavements or rainwater harvesting infrastructure. Effective floodwater management features such as bioswales and rain gardens are absent, and the bowl-like topography which exacerbates stormwater runoff. Water usage efficiency is evident in some locations, like a municipal garden with drip irrigation and drought-resistant plants, but such practices are not widespread. While the Al-Samra Wastewater Treatment Plant recycles a significant portion of wastewater for agricultural use, decentralized greywater recycling systems at the household level are largely lacking.

Flow 7: Energy flow analysis presented in Table S8:

| Energy | Renewable energy solutions | Energy efficient street furniture | Smart grid integration | Energy awareness and management |
| --- | --- | --- | --- | --- |
| Final Score | 2 | 1 | 2 | 2 |

Table S8. Site A – Energy flow analysis

The area relies on traditional lighting with high energy consumption, while Jordan’s overall energy includes 73% from thermal power plants and 26% from renewable sources like wind and solar, with a renewable capacity of approximately 2,445 MW [39]. Despite Jordan’s significant solar potential, there is a lack of energy-efficient street tools. The development of a smart grid system is ongoing, with Phase 1 completed and 2 underway, including advanced metering (AMI) [40], Energy initiatives, such as consumption-based taxes and higher night rates, are in place [39].

Site B, Jabal Amman, Amman.

Figure S9. marked images around Site B presenting the overall atmosphere refferenced in figure S10. (Photographs D & C taken by the author (Anas Tuffaha) in 2023, Photographs A, B, E and F obtained from Google Street View (© Google, 2023), accessed via Google Maps in 2023, available at https://www.google.com/maps/. Imagery was used for academic, non-commercial purposes in accordance with Google’s Terms of Service.)

Flow 1: Biota flow analysis presented in Table S9:

| Biota | Type of Flora and Fauna | General Perspective | Urban Biodiversity | Connectivity | Maintenance & Strategies | Adaptation to Seasons |
| --- | --- | --- | --- | --- | --- | --- |
| Final Score | 2 | 2 | 2 | 2 | 2 | 2 |

Table S9. Site B – Biota flow analysis

Figure S10. Site B google earth aerial photo, followed by the green areas presented in green. (satellite imagery obtained and generated by the author (Anas Tuffaha) using Google Earth Pro (Version 7.3.6.9345, © Google, 2023), available at https://www.google.com/earth/. Imagery was edited for academic visualization purposes using Adobe Photoshop CC 2015 (Version 16.0, © Adobe Inc., https://www.adobe.com/). Used in accordance with Google’s permissions guidelines (http://www.google.com/permissions/geoguidelines/attr-guide.html).)

Features Olive trees, Aleppo Pine, Date palm, Quercus calliprinos (oak), Cupressus Sempervirens (Mediterranean cyprus) and Pinus halepensis (Aleppo pine). The general design incorporates greenery along streets and gathering spaces, with Rainbow Street serving as a pedestrian-friendly axis. Green areas cover about 7% within 1 square kilometer. Connectivity is supported by linear green distributions linked to other neighborhoods through stairs and pathways, with a lack of shade and tree canopies.

Flow 2: Climate flow analysis presented in Table S10:

| Climate | Existing temperature of the neighborhood | Heat Reduction Elements | Building Height | Street Orientation Maximizing shade and wind flow | Microclimate Creation, Modification through design and vegetation |
| --- | --- | --- | --- | --- | --- |
| Final Score | 1 | 1 | 2 | 2 | 1 |

Table S10. Site B – Climate flow analysis

Figure S11. ENVI-met analysis for potential air temperature on 12:00 , 1^st^ of July 24 at 1.5 meter section in zone b.

Despite its proximity to downtown, the area demonstrates slightly cooler temperatures due to reduced asphalt and increased greenery. ENVI-met analysis on July 1st at 12 noon as seen in S11 shows localized cooling in areas with substantial vegetation, where temperatures range from 33°C to 34.3°C, while the median temperature is 37.8°C, and relies on shop shades and awnings prove insufficient during hotter months. Streets are oriented east-west with buildings facing north-south presented in 3d perspectives in figure S12.

Figure S12. The sample area presented in different hours of the day, showcasing shade,heights, and exposure. (3D models were created by the author (Anas Tuffaha) using SketchUp Make 2017 (© Trimble Inc., Version 17.2.2555, https://www.sketchup.com/) and subsequently edited using Adobe Photoshop CC 2015 (© Adobe Inc., Version 16.0, https://www.adobe.com/). All figures were created by the author and are original content.)

Flow 3: Goods flow analysis presented in Table S11:

| Goods | Street Vendors | Sustainable Delivery Systems | Packaging Waste Reduction | Traffic and pollution effects |
| --- | --- | --- | --- | --- |
| Final Score | 2 | 1 | 1 | 2 |

Table S11. Site B – Goods flow analysis

Street vendors are well-organized along the sides of pathways visualized in figure S13, allowing unobstructed movement and fostering social interaction. Traditional delivery methods dominate, like downtown observations. Packaging waste remains a challenge, with frequent use of plastic bags for every purchase. The area experiences significant traffic congestion, while secondary and residential streets surrounding the main road are less congested.

Figure S13. The sectional point of view of the selected area in comparison with the surrounding. (Image obtained from Google Street View (© Google, 2023), accessed via Google Maps in 2023, available at https://www.google.com/maps/. Imagery was used for academic, non-commercial purposes in accordance with Google’s Terms of Service. 3D models were created by the author (Anas Tuffaha) using SketchUp Make 2017 (© Trimble Inc., Version 17.2.2555, <https://www.sketchup.com/)>)

Flow 4: People flow analysis presented in Table S12:

| People | Population density and its impact | Pedestrian flow and comfort | Public safety | Accessibility for all | Social interaction spaces | people stress |
| --- | --- | --- | --- | --- | --- | --- |
| Final Score | 2 | 2 | 2 | 1 | 2 | 2 |

Table S12. Site B – People flow analysis.

Figure S14. The relationship between building height, the people and street widths. (3D models were created by the author (Anas Tuffaha) using SketchUp Make 2017 (© Trimble Inc., Version 17.2.2555, https://www.sketchup.com/) and subsequently edited using Adobe Photoshop CC 2015 (© Adobe Inc., Version 16.0, https://www.adobe.com/). All figures were created by the author and are original content.)

The area has a lower population density than downtown, with around 700 buildings per square kilometer, creating a relaxed and less crowded environment that enhances pedestrian comfort. Pedestrian flow is high and facilitated by wide pavements of at least 2 meters. While the area is accessible, the hilly terrain and inconsistent pavements is an issue as provided in the images within figure S15. Social interaction spaces, including small squares and seating areas that encourage gathering are limited [9,35], finally, Stress levels align with the national stress index [24].

Figure S15. Pedestrian routes followed, the paths taken, and what is obstructing the way. (Image obtained from Google Street View (© Google, 2023), accessed via Google Maps in 2023, available at https://www.google.com/maps/. Imagery was used for academic, non-commercial purposes in accordance with Google’s Terms of Service.)

Flow 5: Waste flow analysis presented in Table S13:

| Wastes | Street Cleaning Schedules | Public Awareness towards littering | Waste segregation systems | Reduction of single use plastics | Waste management infrastructure |
| --- | --- | --- | --- | --- | --- |
| Final Score | 2 | 2 | 2 | 1 | 2 |

Table S13. Site B – Waste flow analysis

The area benefits from regular cleaning and maintenance schedules, with public awareness about littering at 65%, the highest among the surveyed neighborhoods. However, waste segregation systems are minimal, as 63% of residents report never recycling. There are no measures to reduce single-use plastics, with shops distributing plastic bags freely. The waste management infrastructure is somewhat adequate, with large bins placed every 70-80 meters in residential areas [38]. However, public spaces with high footfall lack sufficient bins, leading to occasional littering.

Flow 6: Water flow analysis presented in Table S14:

| Water | Water management elements | Floodwater street elements | Water usage and efficiency | Greywater recycling systems |
| --- | --- | --- | --- | --- |
| Final Score | 2 | 2 | 1 | 1 |

Table S14. Site B – Water flow analysis

The area lacks key water management elements such as rain gardens or water retention basins but benefits from one of the best drainage systems in Amman, directing water effectively to collection zones as seen in the image collection in figure S16. Being situated on a hill, Rainbow Street is less prone to flooding. However, there are no significant water-saving measures like low-flow fixtures or smart irrigation, and greywater recycling systems are entirely absent.

Figure S16. Street drainage in zone B is a method to control rainwater throughout the neighborhood. (Photographs taken by the author (Anas Tuffaha) in 2022,2023)

Flow 7: Energy flow analysis presented in Table S15:

| Energy | Renewable energy and solutions | Energy efficient street furniture | Smart grid integration | Energy awareness |
| --- | --- | --- | --- | --- |
| Final Score | 2 | 1 | 2 | 2 |

Table S15. Site B – Energy flow analysis

The main street offers a walkable environment that reduces reliance on cars and promotes walking, with survey results indicating the highest steps per day among neighborhoods, with most survey respondents mindful of energy management, while only 7% reported no efforts to reduce energy.

Site C, Sweifeyeh, 7th circle, Amman:

Figure S17. marked images around Site C presenting the overall atmosphere refferenced in figure S18. (Image obtained from Google Street View (© Google, 2023), accessed via Google Maps in 2023, available at https://www.google.com/maps/. Imagery was used for academic, non-commercial purposes in accordance with Google’s Terms of Service.)

Flow 1: Biota flow analysis presented in Table S16:

| Biota | Type of Flora and Fauna | General Perspective and Design | Urban Biodiversity Variety | Connectivity | Maintenance & Irrigation Strategies | Adaptation to Seasons |
| --- | --- | --- | --- | --- | --- | --- |
| Final Score | 1 | 2 | 1 | 1 | 1 | 2 |

Table S16. Site C – Biota flow analysis

Figure S18. The sectional point of view of the selected area in comparison with the surrounding. (satellite imagery obtained and generated by the author (Anas Tuffaha) using Google Earth Pro (Version 7.3.6.9345, © Google, 2023), available at https://www.google.com/earth/. Imagery was edited for academic visualization purposes using Adobe Photoshop CC 2015 (Version 16.0, © Adobe Inc., https://www.adobe.com/). Used in accordance with Google’s permissions guidelines (http://www.google.com/permissions/geoguidelines/attr-guide.html).)

dominated by the mediterranean cypress, newly planted Melia azedarach’s, native Phoenix dactylifera, and some Albizia julibrissin, green spaces cover only 1.5% of the area. Green space design focuses on aesthetics, exemplified by Al-Wakalat Street, which features trees along both sides but lacks connectivity to other parts of the neighborhood, limiting its impact. Biodiversity is minimal due to the small green space percentage and limited tree variety. Connectivity is weak, with no significant green infrastructure or "green bridges" linking different zones. Maintenance and irrigation are concentrated on Al-Wakalat Street in red within the figure S22, which comprises of only 5% of the area, leaving other zones poorly maintained with inefficient irrigation systems.

Flow 2: Climate flow analysis presented in Table S17:

| Climate | Existing temperature of street and environment | Heat Reduction Elements | Building Height | Street Orientation by Maximizing shade and wind flow | Microclimate Creation, Modification through design and vegetation |
| --- | --- | --- | --- | --- | --- |
| Final Score | 1 | 1 | 2 | 2 | 1 |

Table S17. Site C – Climate flow analysis

Figure S19. ENVI-met analysis for potential air temperature on 12:00 , 1^st^ of July 24 at 1.5 meter section in zone C.

In Zone C, analyzed on July 1st at 12 noon as seen in figure S20, the median temperature was 35.39°C, influenced by expansive open sandy lots used as parking spaces, which registered between 33°C and 35°C. Al-Wakalat Street offers limited canopy shade from a few trees, while the rest of the area lacks heat-reduction elements like green roofs or shaded walkways. Building heights range from 6–15 meters. Streets are oriented along a southeast grid, with buildings facing north and south. However, there is minimal evidence of microclimate planning, with limited vegetation in courts and gathering spaces.

Figure S20. A simulation of the relation between building orientation and the sun. (3D models were created by the author (Anas Tuffaha) using SketchUp Make 2017 (© Trimble Inc., Version 17.2.2555, https://www.sketchup.com/) and subsequently edited using Adobe Photoshop CC 2015 (© Adobe Inc., Version 16.0, https://www.adobe.com/). All figures were created by the author and are original content.)

Flow 3: Goods flow analysis presented in Table S18:

| Goods | Street Vendors | Sustainable Delivery Systems | Packaging Waste Reduction | Traffic and pollution effects |
| --- | --- | --- | --- | --- |
| Final Score | 2 | 1 | 1 | 1 |

Table S18. Site C – Goods flow analysis

The area features a high concentration of commercial spaces, including a gold market, three malls, and a walkable commercial zone, where vendors are well-integrated, providing local goods without obstructing pedestrian flow, creating natural interaction zones. Goods are delivered using traditional methods, Traffic is a significant issue, with delays exceeding 30 minutes per 10 km.

Flow 4: People flow analysis presented in Table S19:

| People | Population density | Pedestrian flow/comfort | Public safety | Accessibility for all | Social interaction | people stress |
| --- | --- | --- | --- | --- | --- | --- |
| Final Score | 2 | 2 | 2 | 1 | 2 | 2 |

Table S19. Site C – People flow analysis

Figure S21. A solid and void illustration of the Sweifeyeh (Site C) to explain the distribution of buildings. (satellite imagery obtained and generated by the author (Anas Tuffaha) using Google Earth Pro (Version 7.3.6.9345, © Google, 2023), available at https://www.google.com/earth/. Imagery was edited for academic visualization purposes using Adobe Photoshop CC 2015 (Version 16.0, © Adobe Inc., https://www.adobe.com/). Used in accordance with Google’s permissions guidelines (http://www.google.com/permissions/geoguidelines/attr-guide.html).)

The neighborhood has dense urban fabric, with approximately 500 buildings per square kilometer, 32% of which are concrete rooftops as seen in figure S21. This density causes crowding, particularly around commercial zones, affecting residents and visitors. Al-Wakalat Street stands out as a pedestrian-friendly area, but other streets suffer from limited sidewalks, inconsistent shading, and obstructing elements. Public safety is high due to well-lit and maintained commercial streets, though traffic accidents remain a slight risk. Accessibility for disabled individuals is poor due to uneven pavements and a lack of ramps. While organic interaction zones flourish in commercial areas.

Flow 5: Waste flow analysis presented in Table S20:

| Wastes | Street Cleaning Schedules | Public Awareness towards littering | Waste segregation systems | Reduction of single use plastics | Waste management infrastructure design |
| --- | --- | --- | --- | --- | --- |
| Final Score | 2 | 2 | 1 | 1 | 1 |

Table S20. Site C – Waste flow analysis

The area benefits from frequent cleaning schedules, but the high number of retail stores and visitors results in more littering, requiring better control. Public awareness about littering is moderate, with only 48% actively participating in keeping the area clean. Waste segregation systems are absent, and single-use plastics are widely used without regulation. The waste management infrastructure is inadequate, with sufficient bins on the main walkable street but large gaps elsewhere, focused in empty and unbuilt lots in red presented in figure S22 below.

Figure S22. Sweifeyeh (Site C) distribution of bins and waste drop points in red dots, focused on empty lots. (satellite imagery obtained and generated by the author (Anas Tuffaha) using Google Earth Pro (Version 7.3.6.9345, © Google, 2023), available at https://www.google.com/earth/. Imagery was edited for academic visualization purposes using Adobe Photoshop CC 2015 (Version 16.0, © Adobe Inc., https://www.adobe.com/). Used in accordance with Google’s permissions guidelines (http://www.google.com/permissions/geoguidelines/attr-guide.html).)

Flow 6: Water flow analysis presented in Table S21:

| Water | Water management elements | Floodwater street elements | Water usage and efficiency | Greywater recycling systems |
| --- | --- | --- | --- | --- |
| Final Score | 1 | 2 | 1 | 1 |

Table S21. Site C – Water flow analysis

The area lacks visible water management systems, such as rainwater harvesting or permeable pavements, relying entirely on sewer systems for drainage without sustainable management features. The hilly topography naturally directs floodwater to lower zones, preventing flooding, though no formal mitigation systems are in place and there are no greywater recycling systems present in the neighborhood.

Flow 7: Energy flow analysis presented in Table S22:

| Energy | Renewable energy solutions | Energy efficient street furniture | Smart grid integration | Energy awareness and management |
| --- | --- | --- | --- | --- |
| Final Score | 1 | 1 | 1 | 2 |

Table S22. Site C – Energy flow analysis

Sweifeyeh lacks renewable energy solutions such as solar panels or wind turbines, with its two large malls and commercial buildings relying heavily on non-renewable energy from the national grid. Energy-efficient Street furniture is absent, and there is no visible smart grid infrastructure to manage energy dynamically or integrate renewable sources. Survey results indicate moderate energy awareness among residents and business owners.

Site D, Tla’ il-Ali, Amman:

Figure S23. marked images around Site D presenting the overall atmosphere referenced in figure S24. (Image obtained from Google Street View (© Google, 2023), accessed via Google Maps in 2023, available at https://www.google.com/maps/. Imagery was used for academic, non-commercial purposes in accordance with Google’s Terms of Service.)

Flow 1: Biota flow analysis presented in Table S23:

| Biota | Type of Flora and Fauna | General Perspective and Design | Urban Biodiversity Variety | Connectivity | Maintenance & Irrigation Strategies | Adaptation to Seasons |
| --- | --- | --- | --- | --- | --- | --- |
| Final Score | 1 | 1 | 1 | 1 | 1 | 2 |

Table S23. Site D – Biota flow analysis

Figure S24. A solid and void, followed by green areas illustration of the Site D. (satellite imagery obtained and generated by the author (Anas Tuffaha) using Google Earth Pro (Version 7.3.6.9345, © Google, 2023), available at https://www.google.com/earth/. Imagery was edited for academic visualization purposes using Adobe Photoshop CC 2015 (Version 16.0, © Adobe Inc., https://www.adobe.com/). Used in accordance with Google’s permissions guidelines (http://www.google.com/permissions/geoguidelines/attr-guide.html).)

Dominated by Olea europaea, phoenix dactylifera, mediterranean cypress tree, and Quercus coccifera. Additionally, the zone has many old cypress trees, especially near the university, with wild plants on and empty lots like Varthemia iphionoides, green areas make up less than 4% of the neighborhood as seen in figure 34. the northeast side focused on the University of Jordan and the opposite road lined with commercial shops. Biodiversity is limited, and empty land remains unutilized for enhancing green spaces. Connectivity between vegetated areas is poor, discouraging interaction with nature. Maintenance is minimal, with no visible irrigation strategies.

Flow 2: Climate flow analysis presented in Table S24:

| Climate | Existing temperature of street and environment | Heat Reduction Elements | Building Height | Street Orientation by Maximizing shade and wind flow | Microclimate Creation, Modification through design and vegetation |
| --- | --- | --- | --- | --- | --- |
| Final Score | 1 | 1 | 2 | 1 | 1 |

Table S24. Site D – Climate flow analysis

Figure S25. ENVI-met analysis for potential air temperature on 12:00 , 1^st^ of July 24 at 1.5 meter section in zone d.

In Zone D, ENVI-met analysis on July 1st at 12 noon shows the highest median temperature at 36.4°C as seen in figure S25, with consistent temperatures between 36.5°C and 37.5°C. Buildings average 12-15 meters in height, and streets-oriented north to south, with most buildings facing east and west.

Flow 3: Goods flow analysis presented in Table S25:

| Goods | Street Vendors | Sustainable Delivery Systems | Packaging Waste Reduction | Traffic and pollution effects |
| --- | --- | --- | --- | --- |
| Final Score | 2 | 1 | 1 | 1 |

Table S25. Site D – Goods flow analysis

Street vendors in the area are fewer than in other neighborhoods and do not obstruct pedestrian flow. However, there are no sustainable delivery systems or eco-friendly practices for goods transportation, Traffic congestion, particularly on University Street, is a major issue without visible mitigation strategies.

Flow 4: People flow analysis presented in Table S26:

| People | Population density and its impact | Pedestrian flow and comfort | Public safety | Accessibility for all | Social interaction spaces | people stress |
| --- | --- | --- | --- | --- | --- | --- |
| Final Score | 2 | 1 | 2 | 1 | 2 | 2 |

Table S26. Site D – People flow analysis

Figure S26. blocked path throughout site D showcasing blockage of trees and unfixed paves. (Photographs taken by the author (Anas Tuffaha) in 2023.)

The area has moderate population density, with 57-60% built-up space consisting of residential areas and commercial activity along the main street. Traffic congestion is significant, and pedestrian paths are blocked by trees and poorly maintained. While the area is safe, accessibility is low, with limited attention to inclusive design for differently abled individuals. Social interaction spaces are present, including cafes, offices, and commercial areas, in indoor ways, but the overall environment is strained by congestion and pedestrian infrastructure as seen in figure S26.

Flow 5: Waste flow analysis presented in Table S27:

| Wastes | Street Cleaning and maintenance | Public Awareness towards littering | Waste segregation systems | Reduction of single use plastics | Waste management infrastructure design |
| --- | --- | --- | --- | --- | --- |
| Final Score | 2 | 2 | 1 | 1 | 1 |

Table S27. Site D – Waste flow analysis

Street cleaning schedules are regular but lack any special modifications. Public awareness of littering is relatively high compared to other neighborhoods, with 68% of survey respondents indicating proper disposal behavior [Annex A]. However, there are no visible waste segregation methods or efforts to reduce single-use plastics. Waste management infrastructure is basic, with bins distributed at distant intervals, mostly in unused areas, like site C.

Flow 6: Water flow analysis presented in Table S28:

| Water | Water management elements | Floodwater street elements | Water usage and efficiency | Greywater recycling systems |
| --- | --- | --- | --- | --- |
| Final Score | 1 | 2 | 1 | 1 |

Table S28. Site D – Water flow analysis

The neighborhood lacks advanced water management or recycling systems, with no visible features for floodwater management, though the high altitude reduces flooding risks. There are no efficient water usage practices or irrigation systems, and greywater recycling is entirely absent.

Flow 7: Energy flow analysis presented in Table S29:

| Energy | Renewable energy solutions | Energy efficient street furniture | Smart grid integration | Energy awareness |
| --- | --- | --- | --- | --- |
| Final Score | 1 | 1 | 1 | 2 |

Table S29. Site D – Energy flow analysis

The neighborhood lacks renewable energy systems, energy-efficient street furniture, and smart grid integration. However, survey responses indicate that residents are highly aware of energy conservation both at home and in public spaces, which may be linked to the large number of residential properties (Appendix A).

Appendix C, Survey:

• What is your age category ?

under 24

25-34

35-54

55+

• which location or area fits your residence?

1. Downtown

2. Jabal Amman

3. Al sweifeyeh, 7^th^ circle

4. Tla’ Al-Ali

5. Other

• Do you feel the people in your neighborhood are aware of street cleanliness and throwing trash correctly ?

Yes the majority does

Some people do

No

• When it comes to waste segregation ( Recycling ) is your neighborhood aware and active towards it ?

Yes the majority does

Some people do

No

• Regarding Energy Awareness, are there efforts and public awarness to use energy effecient tools like solar energy?

Yes the majority does

Some people do

No

• In your neighborhood which of these is the biggest problem and solving it could improve your neighborhoods drastically ?

Lack of green areas or vegitation

Climate control and extreme weather

The People lack interaction, ( no correct interaction zones or methods)

Wastes, cleanliness

Water flooding and management, water shortage

Increased Traffic

Street vendors

High energy costs, lack of sustainable energy

• check the category that needs devolopment to improve your local street to improve the daily live's of the locals?

Increase walkable area

Climate control and extreme weather mitigation.

add street elements (like seats), and interaction zones.

Recycling bins and waste disposal awareness

Water flooding, management & shortage

improve parking spots, car/people relationship, reduce traffic

Add energy effecient systems like solar power

• on average how many steps or Km's do you walk per day ?

0-3000 (Under 2.5km)

3000-6000 (2.5km-5km)

6000-10000 (5km-8km)

10000 + (8km +)

I don’t keep track

Appendix D, Multivariate Regression Analysis:

To identify causal relationships between metabolic flows and pedestrian walkability, a multivariate linear regression model was applied, controlling for socio-economic variables.

Dependent VariableS:

Walkability Score (standardized 10-point scale).

Independent Variables:

Biota Score (10-point scale).

Goods Flow Score (traffic congestion, 10-point scale).

Climate Score (ENVI-met temperature deviation).

Income Level (survey-derived ordinal scale: 1 = low, 5 = high).

Regression Results

| Variable | Coefficient (β) | Std. Error | p-value | 95% Confidence Interval |
| --- | --- | --- | --- | --- |
| Biota Score | 0.69 | 0.12 | <0.001 | [0.48, 0.90] |
| Goods Flow Score | -0.58 | 0.18 | 0.007 | [-0.89, -0.27] |
| Climate Score | -0.21 | 0.09 | 0.061 | [-0.39, 0.02] |
| Income Level | 0.33 | 0.15 | 0.043 | [0.06, 0.60] |
| R² | 0.82 |  |  |  |

Table S30. Multivariate Regression Results for Walkability Predictors.

Interpretation:

Biota Score: A 1-point increase in biota score corresponds to a 0.69-point increase in walkability (p < 0.001), confirming greenery’s critical role.

Goods Flow: A 1-point increase in traffic congestion (lower goods score) reduces walkability by 0.58 points (p = 0.007).

Income Level: Higher-income neighborhoods showed 0.33-point higher walkability (p = 0.043), aligning with observed infrastructure disparities.

**Identifiable individuals statement:**

This study does not include any identifiable information or images of human participants.
